# Large-scale metabolic interaction network of the mouse and human gut microbiota

**DOI:** 10.1038/s41597-020-0516-5

**Published:** 2020-06-26

**Authors:** Roktaek Lim, Josephine Jill T. Cabatbat, Thomas L. P. Martin, Haneul Kim, Seunghyeon Kim, Jaeyun Sung, Cheol-Min Ghim, Pan-Jun Kim

**Affiliations:** 1 Department of Biology, Hong Kong Baptist University, Kowloon, Hong Kong; 2 School of Life Sciences, Ulsan National Institute of Science and Technology, Ulsan, 44919 Republic of Korea; 3 Analytics, Computing, and Complex Systems Laboratory, Asian Institute of Management, Makati, 1229 Metro Manila Philippines; 4 Department of Physics, Ulsan National Institute of Science and Technology, Ulsan, 44919 Republic of Korea; 5 Department of Physics, Pohang University of Science and Technology, Pohang, Gyeongbuk 37673 Republic of Korea; 6 Samsung SDS, Seoul, 05510 Republic of Korea; 7 Microbiome Program, Center for Individualized Medicine, Mayo Clinic, Rochester, MN 55905 USA; 8 Division of Surgical Research, Department of Surgery, Mayo Clinic, Rochester, MN 55905 USA; 9 Division of Rheumatology, Department of Internal Medicine, Mayo Clinic, Rochester, MN 55905 USA; 10 Department of Molecular Pharmacology and Experimental Therapeutics, Mayo Clinic, Rochester, MN 55905 USA; 11 Center for Quantitative Systems Biology, Hong Kong Baptist University, Kowloon, Hong Kong; 12 Institute of Computational and Theoretical Studies, Hong Kong Baptist University, Kowloon, Hong Kong; 13 Abdus Salam International Centre for Theoretical Physics, 34151 Trieste, Italy

**Keywords:** Biochemical networks, Microbial ecology, Literature mining, Microbiome

## Abstract

The role of our gut microbiota in health and disease is largely attributed to the collective metabolic activities of the inhabitant microbes. A system-level framework of the microbial community structure, mediated through metabolite transport, would provide important insights into the complex microbe-microbe and host-microbe chemical interactions. This framework, if adaptable to both mouse and human systems, would be useful for mechanistic interpretations of the vast amounts of experimental data from gut microbiomes in murine animal models, whether humanized or not. Here, we constructed a literature-curated, interspecies network of the mammalian gut microbiota for mouse and human hosts, called NJC19. This network is an extensive data resource, encompassing 838 microbial species (766 bacteria, 53 archaea, and 19 eukaryotes) and 6 host cell types, interacting through 8,224 small-molecule transport and macromolecule degradation events. Moreover, we compiled 912 negative associations between organisms and metabolic compounds that are not transportable or degradable by those organisms. Our network may facilitate experimental and computational endeavors for the mechanistic investigations of host-associated microbial communities.

## Abstract (summary)

**Table:** 

Measurement(s)	metabolic process • transport • macromolecule catabolic process • gut microbiome measurement
Technology Type(s)	digital curation • Phylogenetic Analysis
Factor Type(s)	Species of microorganisms • Type of host cells
Sample Characteristic - Organism	Homo sapiens • Mus musculus • Bacteria • Archaea • Eukaryota

Machine-accessible metadata file describing the reported data: 10.6084/m9.figshare.12336164

## Background & Summary

The mammalian intestinal tract is colonized by various microorganisms, called the gut microbiota or microbiome^1–3^. Recent advances in metagenomics have revealed that alterations in the human gut microbiota are implicated in a number of disorders, such as obesity, inflammatory bowel disease, colorectal cancer, and diabetes^4–7^. At the center of the gut microbiota functions are the various interactions between microbes and their interplay with the host environment^2,6,8^. Microbes degrade diet-derived and host-derived chemical substances, and release the degradation products to other members of the community. The microbial transport of nutrients and metabolic byproducts gives rise to competition for resources and cooperative relationships via metabolic cross-feeding^2,8^. The metabolites secreted by the microbes are absorbed by host tissues, and translate into beneficial or detrimental mediators of host physiology^6,9^. As a result, such microbe-microbe and microbe-host interactions form a complex ecological network in the gut environment^10^.

In the microbiome research, one common practice for reconstructing metabolite-mediated microbial networks is to combine the entire biochemical reactions inferred from annotated metagenomes^11,12^. This method, by its nature, does not delineate biochemical reactions to the species from which they originate, making it difficult to elucidate interspecies interactions. On the other hand, there exist previous works on the modeling of diverse interspecies interactions explicitly mediated by metabolites that are transported (imported or exported) by individual microbial species^13,14^. Yet, these works are based on error-prone, automated identification protocols for transportable metabolites, which are possibly inaccurate to some degrees. There are ongoing computational efforts towards biologically realistic microbial interactions, by using manually curated, constraint-based metabolic models or relatively simple kinetic models^15,16^. Nevertheless, most of these models are far from the scale of diversity seen in the gut community, which typically comprises hundreds of different microbial species. Notably, this scale of microbial diversity has been recently captured by constraint-based metabolic models with semi-automatic model reconstructions^17^, but they still exhibit limited biological accuracies^18–20^.

Recently, we have constructed an extensive, literature-curated interspecies metabolic interaction network of the human gut microbiota, NJS16, which represents another system-level framework for gut microbiota analysis^10^. This network is primarily based on biological knowledge and experimental evidence documented in the literature. The network NJS16 encompasses >4,000 small-molecule transport and macromolecule degradation events of >500 bacterial and archaeal species and 3 human cell types. Although NJS16 is useful to explore the microbial community inside the human gut, mechanistic studies in the microbiome research field have been mainly conducted on animal models, rather than on human subjects, due to the technical and regulatory limitations on human experimentation^21,22^. Regarding animal models, physiological, anatomical, and genetic similarities between humans and mice, as well as massively accumulated knowledge of mouse genetics, have facilitated the use of murine models, to elucidate causality and mechanisms of host-microbiota interactions^4,7,23^. In this regard, a phylogenetic extension of NJS16 to murine gut microbes would be useful for the system-level mechanistic exploration of gut microbiota functions using murine animal models.

**Fig. 1:**
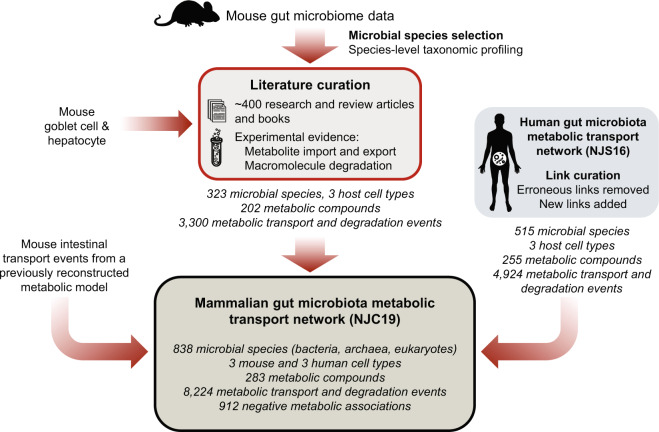
Construction of the mammalian (mouse and human) gut microbiota interaction network NJC19. The flow chart of the network construction is presented. NJC19 is mainly built upon literature-curated, metabolic information of the mouse gut microbiota, combined with the revised version of NJS16 that represents the human gut microbiota interaction network.

Here, we present a literature-curated, interspecies metabolic interaction network of the microbiota associated with the mouse and human gut, NJC19. To our knowledge, NJC19 represents the largest ever, literature-based network data resource for the mammalian gut microbiota, as a compilation of information from 769 research and review articles and textbooks (Fig. 1). This network is an advancement from our previous network, NJS16, which is limited to the human gut microbiota^10^. Specifically, NJC19 greatly expands the diversity of microbial species and host cells to those relevant to the mouse gut environments, and even covers a certain range of eukaryotic microbes that were completely missing in the predecessor NJS16. Therefore, NJC19 serves as a global network template, adaptable to the gut microbiota of either a mouse, human, or humanized mouse. Moreover, not only does NJC19 incorporate metabolite transport and macromolecule degradation events of the microbiota, but it also provides literature-annotated, *negative* information of which metabolic compounds are not able to be transported or degraded by the organisms. Such negative information would be useful to curate computational microbial models, such as constraint-based metabolic models, which can include false-positive transport reactions from automatic genome annotations.

We expect our network NJC19 to be a useful template for the mechanistic interpretation of various microbiome data from murine and human experiments.

## Methods

### Collection of mouse microbiome data and taxonomic identification for NJC19 construction

We aimed to construct a large-scale network for the mammalian gut microbiota that comprises microbial species populating the mouse and human gut. Figure 1 provides the overview of our network construction procedure. To construct the network, we started by collecting raw shotgun metagenome and 16S rRNA gene sequence data from fecal and cecal samples of laboratory and wild-caught mice from seven different studies^3,24–29^, as detailed in Online-only Table 1. It is noteworthy that the inclusion of the data from wild-caught mice^3^ allows the coverage of diverse microbial communities associated with natural murine lifestyles. The species-level taxonomic profiling of the shotgun metagenome sequence data was performed using the MetaPhlAn v2.0 software, which utilizes clade-specific marker sequences to identify microbial taxa^30^. When using MetaPhlAn v2.0, the “sensitive-local” mapping option was selected. For the taxonomic profiling of 16S rRNA gene sequence data, we used the open-reference OTU picking workflow of QIIME v1.8.0 with Greengenes v13_8_pp reference files^31^, and then selected species-level microbial taxa from the results. Among all species detected from the metagenome and 16S rRNA gene sequence data, priority for the collection of metabolic information (see below) was given to species absent in our previous network, NJS16^10^. In the case of the metagenome sequence data, the number of the detected species was rather excessive for our further processing; therefore, among those species, we only considered the species inhabiting ≥90% of the metagenome samples (with the relative abundance ≥0.001%) in each study. We found that the genera of these selected species account for the vast majority [89.6 ± 4.3% (avg. ± s.d.)] of the total microbial abundances in the metagenome samples. In addition, we manually considered some relevant species, such as *Citrobacter rodentium*^32^ (Online-only Table 2–3).

### Collection and integration of metabolic information for NJC19 construction

Using the repertoire of the aforementioned microbial species, metabolic information primarily collected for NJC19 construction was direct experimental evidence of the import and export of small-molecule metabolites (e.g., sugars, vitamins, organic acids, and gases) and the degradation of macromolecules (e.g., starch, cellulose, hemicellulose, and mucin), reported in literature. For the small-molecule metabolites, we mostly considered primary metabolites, i.e., nutrients and metabolic byproducts associated with microbial growth or reproduction. In addition, literature sources that report the mRNA or protein expression for metabolite-specific enzymes or transporters were considered. When encountering the information of which chemical compounds are not able to be transported or degraded by a given organism, we recorded this negative information as well, as part of our collected data. Despite technically not being a part of the gut microbiota, some host cells directly affect or are affected by microbial metabolism, and thus were considered to be a functional extension of the microbial community. In a similar fashion to our previous network NJS16 for the human case, the specific mouse tissue cells that we considered were the intestinal absorptive cell, the mucin-secreting goblet cell, and the bile acid-secreting hepatocyte. Although the hepatocyte is not part of intestinal tissue, its secreted bile acids are utilized by microbes in the gut. In the case of the mouse intestinal absorptive cell, the information from a manually-curated, genome-scale metabolic model (iSS1393) was adopted^33^. All annotated metabolite transport or macromolecule degradation processes for different strains of the same species were consolidated for that species as its collective feature. Because degradation of a given macromolecule is often performed by multiple species in the gut, we considered the corresponding degradation products to be indirect export products of all species participating in that macromolecule degradation. In this work, we differentiated two macromolecules, xylan and mannan, from a “hemicellulose” macromolecule in NJS16, for more specific representation of their degradation products.

In parallel, we carefully re-examined the existing components of NJS16^10,34^, and removed the incorrectly-placed components and added new links found from literature, according to more specific and accurate information. The revised NJS16 was finally connected to the above mouse gut microbiota interaction network through the common chemical compounds shared by the both networks, to form the mouse and human gut microbiota interaction network, NJC19 (Fig. 1 and Table 1). NJC19 is provided in both human- and machine-readable forms, through Online-only Tables 1–5(XLSX files) and JavaScript Object Notation (JSON) files deposited in the Dryad Digital Repository^35^, respectively. In addition, the Cytoscape Session (cys) file of NJC19 is provided for interactive network visualization^35^.

**Table 1:** Datasets used for the construction and validation of NJC19.

Source	Processing	Data
Mouse fecal and cecal microbiome data (Online-only Table 1).	Application of taxonomic analysis tools to the microbiome data (Methods).	Output flies of the taxonomic analysis tools, which include the lists of identified microbial taxa and their relative abundances^35^.
Lists of identified microbial taxa and their relative abundances from mouse fecal and cecal microbiome data^35^.	Selection of microbial species in metagenome samples based on the frequency of the species occurrence across the samples (Methods).	List of selected microbial species in the metagenome samples^35^.
Literature (Online-only Table 2) and NJS16^34^.	Manual collection of metabolic information from literature, and revision of NJS16 (Methods).	Interaction network of microbial species/host cells mediated by metabolic compounds, NJC19 (Online-only Tables 3–5 and the corresponding JSON files^35^).
Mouse microbiome and metabolome data^4,37,38^.	Extraction of taxonomic compositions and metabolite levels (see Technical Validation and Fig. 3 legend).	Taxonomic compositions^35^, and microbial producer and metabolite levels for NJC19 validation (Fig. 3).

## Data Records

Our network NJC19 offers the reference map of the mammalian gut microbiota and chemical compound relationships (from 769 literature sources), which can be adapted for each context of mouse, human, and humanized mouse microbiomes. In NJC19, one set of nodes corresponds to organisms (i.e., microbial species and host cells), while the other set corresponds to chemical compounds (i.e., small-molecule metabolites or macromolecules). An organism and a chemical compound are connected if the organism imports, exports, or degrades the chemical compound. NJC19 comprises 838 microbial species (766 bacteria, 53 archaea, and 19 eukaryotes) in the mouse and human gut, 6 mouse and human cell types metabolically interacting with those microbes, and 283 chemical compounds (266 small molecules and 17 macromolecules)—all interconnected by 8,224 small-molecule transport or macromolecule degradation events. In addition, NJC19 provides information on small molecules and macromolecules that are reportedly not transportable or degradable by certain organisms—described through 912 negative metabolic associations. These negative associations can be particularly useful for the curation of automatically-generated metabolic models, which may include false-positive transport reactions derived from inaccurate genome annotations.

**Fig. 2:**
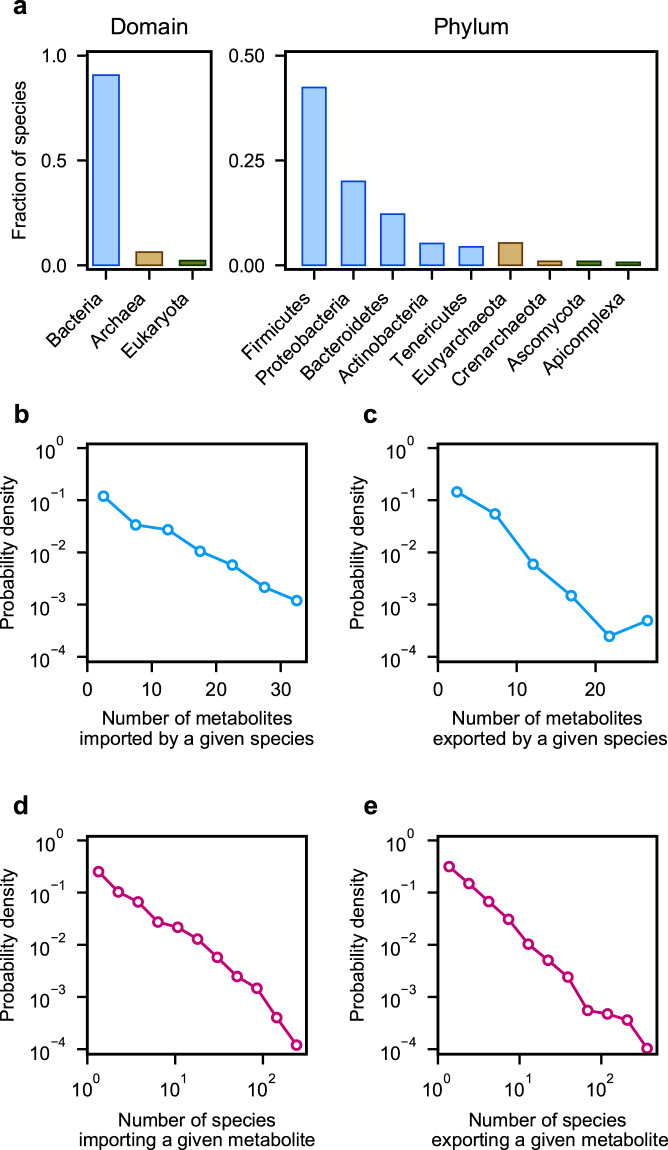
Microbial taxonomic composition and network structural properties of NJC19. **(a)** Fraction of microbial species in the network, which belong to each domain (left) or phylum (right). The right panel shows several phyla with the largest fractions in each domain. Both left and right panels show bacteria in blue, archaea in tan, and eukaryotes in green. (**b**,**c**) The vertical axis represents the distribution of the probability *P*(*k*) that a given microbial species imports (**b**) or exports (**c**) *k* metabolites on the horizontal axis. (**d**,**e**) The vertical axis represents the distribution of the probability *P*(*k*) that a given metabolite is imported (**d**) or exported (**e**) by *k* species on the horizontal axis.

Figure 2a shows the overall phylogenetic composition of microbial species included in NJC19. To overview the network topology of NJC19, we counted the number of metabolites imported or exported by each microbial species. Each species in the network imports 5.8 and exports 3.5 metabolites on average, and the probability that a given species imports (or exports) *k* metabolites follows an exponential distribution *P*(*k*) ∝ *e*^−*rk*^ (*r* ≈ 0.2 and 0.3 for the import and export cases, respectively; see Fig. 2b,c). *Bacteroides thetaiotaomicron* is one of the most promiscuous species, importing 33 and exporting 29 metabolites. Conversely, for each metabolite, we counted the number of species importing or exporting that metabolite. The probability that a given metabolite is imported (or exported) by *k* species follows a power-law distribution *P*(*k*) ∝ *k*^−*γ*^ (*γ* ≈ 1.4 for both import and export cases; Fig. 2d,e), which is much broader than the above exponential distributions. Among metabolites, glucose and acetate are the most frequent substrate and product, respectively, and are imported by 303 species (36.2% of the total species) and exported by 461 species (55.0% of the total species). In contrast, an average metabolite is imported by 21.8 species and exported by 13.0 species. Collectively, metabolites are highly uneven in terms of the ranges of their transporting species.

As noted above, the full details of NJC19 are available in both human- and machine-readable forms, through Online-only Table 1 and JSON files in the Dryad Digital Repository^35^, respectively. As noted above, the cys file of NJC19 is available for network visualization^35^, and can be accessed by Cytoscape v3.7.2^36^.

Online-only Table 1 shows the detailed sources of mouse metagenome and 16S rRNA gene sequence data that were used for microbial species identification when we constructed NJC19. Online-only Table 2 shows the literature sources of metabolic information used for NJC19 construction. Online-only Table 3 shows the list of microbial species and host cell types in NJC19. The name of each microbial species is presented with the NCBI taxonomy ID. Online-only Table 4 includes the list of small-molecule metabolites and macromolecules in NJC19. The name of each compound is presented with the KEGG compound ID. Supplementary Table 1 provides all the metabolic associations between chemical compounds and microbial species/host cells in NJC19, along with their literature sources. These metabolic associations include both positive and negative associations (see above). Online-only Table 5 shows the degradation products of macromolecules in NJC19.

On the other hand, our JSON files^35^ include “NJC19_network.json”, “NJC19_organism.json”, “NJC19_compound.json”, and “NJC19_reference.json”. Among them, “NJC19_network.json” is equivalent to in Supplementary Table 1, in terms of its contents. This file consists of a total of 9,136 items. Each object in the file is exactly matched with one association in Supplementary Table 1. Each object has its own identification number that starts with “NJC19_” followed by a five-digit number. The object includes four key-value pairs. The keys are “Species”, “Small-molecule metabolite or macromolecule”, “Metabolic activity”, and “Ref. #”, reminiscent of the column names in Supplementary Table 1. The other files “NJC19_organism.json”, “NJC19_compound.json”, and “NJC19_reference.json” include detailed information on the values of the keys “Species”, “Small-molecule metabolite or macromolecule”, and “Ref. #” in the file “NJC19_network.json”, respectively. In a similar fashion to Online-only Tables 3 and 4, each microbial species in “NJC19_organism.json” is annotated with the NCBI taxonomy ID, and each compound in “NJC19_compound.json” is annotated with the KEGG compound ID. Furthermore, the specific sample sources of these microbial species are also present in “NJC19_organism.json”. Full metadata of these JSON files are provided in another file “README_NJC19.txt”, which is available in the Dryad Digital Repository together with the JSON files^35^.

As described in Methods, our NJC19 construction was started with taxonomic identification of mouse gut microbiome samples. The comprehensive repertoire of those microbial taxa, identified before the collection of their metabolic information, is provided in the Dryad Digital Repository^35^ (Table 1). In the case of the metagenome samples, it also provides the list of the selected species based on the frequency of their occurrence across the samples (Methods).

## Technical Validation

Metabolic information collected in this study was primarily experimental evidence of small molecule transport and macromolecule degradation events, reported in the literature. Given the information dispersed across research papers, review articles, and textbooks (Online-only Table 2), a careful read of these sources was done to distinguish experimentally-verified information from the predictions solely based on automated bioinformatics algorithms. To check the accuracy of our network, the entire individual links in the compiled network were thoroughly re-examined by the independent authors who had not participated in the initial construction of the network. If potential errors were identified from the examined links (e.g., errors from the possible misinterpretations of the literature), these errors were carefully corrected based on the discussion of multiple authors.

**Fig. 3:**
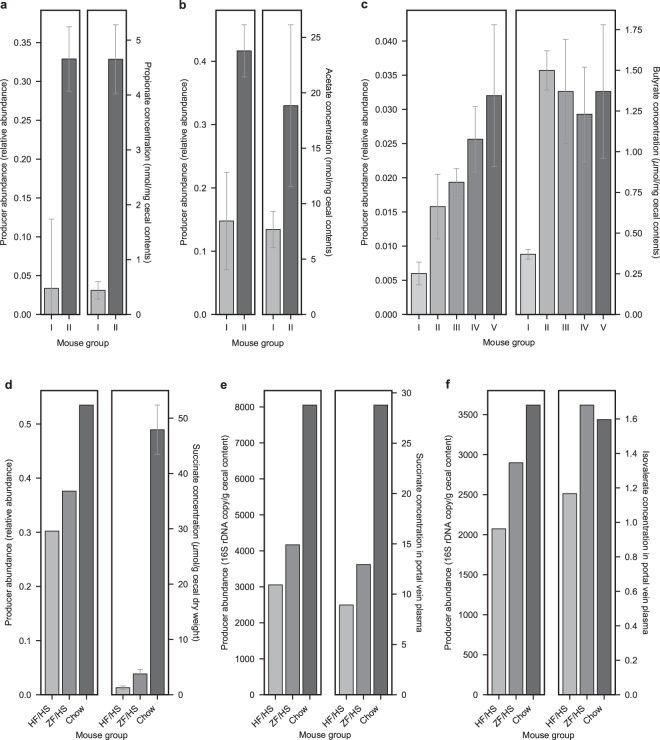
Comparison of mouse microbiome and metabolome data based on NJC19. (**a**,**b**) In the left panels, the abundances of the propionate (**a**) and acetate (**b**) producers in NJC19 were obtained from cecal 16S rRNA gene sequence data^35^ in ref. ^37^ Cecal metabolite concentrations in the right panels were obtained from Fig. 3c of the same study. Mouse group I consists of mice six weeks after 10-day cefoperazone treatment on mouse group II, cefoperazone-naive mice. (**c**) In the left panel, the abundances of the butyrate producers in NJC19 were obtained from fecal 16S rRNA gene sequence data^35^ in ref. ^4^ Cecal butyrate concentrations in the right panel were obtained from Fig. 2c of the same study. Although we used the fecal 16S rRNA gene sequence data for the left panel due to the limited data availability, fecal and cecal microbial compositions were found to strongly correlate in other samples from that study, allowing us to use the fecal sequence data as a proxy for the cecal ones. All mice were initially germ-free in the study. Mouse groups I to V comprise mice transplanted with an obese twin’s microbiota (Ob; group I), mice co-housed with Ob and Ln mice (group II; see next for the definition of Ln mice), mice transplanted with the lean co-twin’s microbiota (Ln; group III), Ob mice co-housed with Ln and germ-free mice (group IV), and Ln mice co-housed with Ob and germ-free mice (group V). (**d**–**f**) In the left panels, the abundances of the succinate (**d**,**e**) and isovalerate (**f**) producers in NJC19 were obtained from cecal 16S rRNA gene copy levels in Fig. 2a of ref. ^38^ We re-scaled succinate producers in (**d**) to the total 16S rRNA gene copy levels, because the corresponding succinate concentrations (**d**) were available per cecal dry weight (i.e., per cecal microbial load). Cecal and portal vein metabolite concentrations in the right panels were obtained from Fig. 2b and Supplementary Table 4 of the same study, respectively. Mouse groups ‘HF/HS’, ‘ZF/HS’, and ‘Chow’ represent gnotobiotic mice on a high-fat/high-sucrose diet, a zero-fat/high-sucrose diet, and a chow diet, respectively. In (**a**–**d**), each error bar represents standard deviation across replicates. Error bars are missing for (**e**,**f**), as well as for the left panel in (**d**), and units are missing for the right panels in (**e**,**f**), because of the information unavailability from the data sources.

To further assess the validity of our network, we examined the correlations between microbe-metabolite links in the network and measured metabolite levels in the mouse gut and portal vein plasma. Specifically, we examined whether the abundance increase/decrease of microbes associated with a particular metabolite in our network is consistent with the shift of the metabolite level across different experimental conditions. Regarding this analysis, three published mouse studies were found to provide the information of both microbial and metabolite levels in their collected samples: one study is for antibiotics (cefoperazone) treatment and recovery^37^, another is for fecal microbiota transplantation from twins discordant for obesity^4^, and the other for gnotobiotic mice with multiple diets^38^. From these studies, we considered only the cases with clear variations in the microbial and metabolite levels, which span at least 1.5-fold changes across different mouse groups for the metabolites and their microbial producers/consumers in NJC19. We further excluded host- and diet-derived metabolites, which may confound our analysis focusing on the effects of microbial metabolism. For all the resulting metabolites, Fig. 3 presents the levels of their microbial producers in NJC19 and those metabolite levels across the mouse groups with varying experimental conditions (Table 1). In Fig. 3, we did not consider microbial consumers because they were relatively deficient in their abundance, less than a half of the producers in each case. Here, microbial producers of each metabolite from the gnotobiotic mouse study^38^ in Fig. 3 are defined as the microbial species that produce this metabolite in NJC19. However, for the other two studies in Fig. 3, the finest taxonomic information is available at the genus level^35^, from the 16S rRNA gene sequence data processed by the Ribosomal Database Project Classifier in this analysis (RDP Naive Bayesian rRNA Classifier Version 2.11 with 16S rRNA training set 16)^39^. Therefore, for these two studies, microbial producers of a given metabolite are defined as the microbial genera, with each having the species whose majority (>50%) can produce that metabolite in NJC19. We also defined microbial consumers in a similar way, although they were excluded from Fig. 3 as discussed above.

Figure 3 indeed demonstrates that alternations in the producer and metabolite levels tend to agree with each other, with the overall 71.4% matches of their increasing or decreasing tendencies across the mouse groups. For example, the propionate producers in NJC19 decreased by 90.0% in the cecum after cefoperazone treatment and recovery, consistent with an 88.5% decrease in the cecal propionate concentration (Fig. 3a). Likewise, the acetate producers in NJC19 decreased by 64.5% at the same time, consistent with a 59.3% decrease in the cecal acetate concentration (Fig. 3b). In these examples, the total microbial loads remained similar during the experiments^37^, and thus the metabolite concentration changes here are not likely to be a mere consequence of the microbial load changes. To test the statistical significance of these correlations, we introduce quantities *f*_*ij*_, *g*_*ij*_, and *f*_*ij*_’ for each pair of mouse groups *i* and *j*: *f*_*ij*_ (*g*_*ij*_) denotes a fold change from group *i* to group *j* in the measured producer (metabolite) abundance averaged over the replicates in the group *i* or *j*. *f*_*ij*_’ denotes a group-*i*-to-*j* fold change in the average abundance of randomly-assigned producers, while the number of those randomly-assigned producers from all the data sources in Fig. 3 is maintained as the same as the number of the total observed producers. To assess the significance of the observed producer and metabolite correlations against a scenario that the producer information in NJC19 may not be more correct than expected by chance, we computed the *P* value as the probability of satisfying *f*_*ij*_’ ≥ *f*_*ij*_ (*f*_*ij*_’ ≤ *f*_*ij*_) for all (*i*, *j*)-pairs that have *f*_*ij*_ and *g*_*ij*_ with both ≥ 1 (≤1). Accordingly, our *P* value calculation reveals that the producers in NJC19 and the detected metabolic compounds are significantly correlated in Fig. 3, thereby supporting the validity of the microbe-compound associations in NJC19 (*P* = 0.02 for Fig. 3a,c–e, *P* < 10^–4^ for Fig. 3f, and *P* = 0.06 for Fig. 3b).

## Supplementary information

Supplementary Table

## Data Availability

Our Python code that converts the JSON format of NJC19 network data (NJC19_network.json^35^) to the format of Supplementary Table 1 can be downloaded from the Dryad Digital Repository^35^. For the taxonomic profiling of microbiome samples for the NJC19 construction, we used MetaPhlAn v2.0 with the “sensitive-local” mapping option and QIIME v1.8.0 with Greengenes v13_8_pp reference files^30,31^, as described above. The aforementioned cys file of NJC19 for network visualization was produced by Cytoscape v3.7.2^36^.

## Online-only Table

**Online-only Table 1:** Sources of mouse microbiome samples for microbial species identification for NJC19 construction.

- *Shotgun metagenome sequence data*				
Reference	Mouse strains	Sample source	No. of samples	Data source
Wang J, Linnenbrink M, Künzel S, Fernandes R, Nadeau M-J, Rosenstiel P et al. Dietary history contributes to enterotype-like clustering and functional metagenomic content in the intestinal microbiome of wild mice. Proc Natl Acad Sci U S A. 2014;111:E2703–10.	wild-caught mice	stool	26	Kindly provided by the authors' group
Pickard JM, Maurice CF, Kinnebrew MA, Abt MC, Schenten D, Golovkina TV et al. Rapid fucosylation of intestinal epithelium sustains host–commensal symbiosis in sickness. Nature. 2014;514:638–41.	(B6.129X1-Fut2tm1Sdo/J) mice backcrossed greater than 7 generations to BALB/c	stool	12	GSE60874
Cullender TC, Chassaing B, Janzon A, Kumar K, Muller CE, Werner JJ et al. Innate and adaptive immunity interact to quench microbiome flagellar motility in the gut. Cell Host Microbe. 2013;14:571–81.	C57BL/6 (TLR5−/− and wild-type)	cecum	10	mgp6393
Langille MG, Meehan CJ, Koenig JE, Dhanani AS, Rose RA, Howlett SE et al. Microbial shifts in the aging mouse gut. Microbiome. 2014;2:50.	C57BL/6	stool	21	mgp3907
Rooks MG, Veiga P, Wardwell-Scott LH, Tickle T, Segata N, Michaud M et al. Gut microbiome composition and function in experimental colitis during active disease and treatment-induced remission. ISME J. 2014;8:1403–17.	BALB/c T-bet-/- Rag2-/-	stool	6	mgp6698
***- 16S rRNA gene sequence data***				
**Reference**	**Mouse strains**	**Sample source**	**No. of samples**	**Data source**
Benson AK, Kelly SA, Legge R, Ma F, Low SJ, Kim J et al. Individuality in gut microbiota composition is a complex polygenic trait shaped by multiple environmental and host genetic factors. Proceedings of the National Academy of Sciences. 2010;107:18933–8.	C57BL/6 and ICR-derived HR intercross line	stool	645	UNL Core for Applied Genomics and Ecology
Wang J, Linnenbrink M, Künzel S, Fernandes R, Nadeau M-J, Rosenstiel P et al. Dietary history contributes to enterotype-like clustering and functional metagenomic content in the intestinal microbiome of wild mice. Proc Natl Acad Sci U S A. 2014;111:E2703–10.	wild-caught mice	stool	66	ERP004395
Linnenbrink M, Wang J, Hardouin EA, Künzel S, Metzler D, Baines JF. The role of biogeography in shaping diversity of the intestinal microbiota in house mice. Mol Ecol. 2013;22:1904–16.	house mice	cecum	201	ERP001970
Pickard JM, Maurice CF, Kinnebrew MA, Abt MC, Schenten D, Golovkina TV et al. Rapid fucosylation of intestinal epithelium sustains host–commensal symbiosis in sickness. Nature. 2014;514:638–41.	(B6.129X1-Fut2tm1Sdo/J) mice backcrossed greater than 7 generations to BALB/c.	stool	14	mgp10494
Rooks MG, Veiga P, Wardwell-Scott LH, Tickle T, Segata N, Michaud M et al. Gut microbiome composition and function in experimental colitis during active disease and treatment-induced remission. ISME J. 2014;8:1403–17.	BALB/c T-bet−/−, Rag2−/−	stool	154	mgp6698

**Online-only Table 2:** List of literature sources of metabolic information used for NJC19 construction.

Ref. #	First author	Year	Title
1	Fontes	2010	Cellulosomes: highly efficient nanomachines designed to deconstruct plant cell wall complex carbohydrates
2	Lynd	2002	Microbial cellulose utilization: fundamentals and biotechnology
3	Patel	1980	Isolation and Characterization of an Anaerobic, Cellulolytic Microorganism, Acetivibrio cellulolyticus gen. nov., sp. nov.
4	Illeghems	2013	Complete genome sequence and comparative analysis of Acetobacter pasteurianus 386B, a strain well-adapted to the cocoa bean fermentation ecosystem
5	Oren	2002	Halophilic Microorganisms and their Environments (Chapter 4)
6	Lazarev	2011	Complete Genome and Proteome of Acholeplasma laidlawii
7	Kay	2001	Recurrent achromobacter piechaudii bacteremia in a patient with hematological malignancy
8	Kiredjian	1986	Alcaligenes piechaudii, a New Species from Human Clinical Specimens and the Environment
9	Reverdy	1984	Nosocomial colonization and infection by Achromobacter xylosoxidans
10	Rogosa	1969	Acidaminococcus gen. n., Acidaminococcus fermentans sp. n., Anaerobic Gram-negative Diplococci Using Amino Acids as the Sole Energy Source for Growth
11	Chang	2010	Complete genome sequence of Acidaminococcus fermentans type strain (VR4T)
12	Eschenlauer	2002	Ammonia Production by Ruminal Microorganisms and Enumeration, Isolation, and Characterization of Bacteria Capable of Growth on Peptides and Amino Acids from the Sheep Rumen
13	Jumas-Bilak	2007	Acidaminococcus intestini sp. nov., isolated from human clinical samples
14	Clark	1995	Acidimicrobium ferrooxidans gen. nov., sp. nov.: mixed-culture ferrous iron oxidation with Sulfobacillus species
15	Kuseel	1999	Microbial Reduction of Fe(III) in Acidic Sediments: Isolation of Acidiphilium cryptum JF-5 Capable of Coupling the Reduction of Fe(III) to the Oxidation of Glucose
16	Zhou	2007	Isolation of a strain of Acidithiobacillus caldus and its role in bioleaching of chalcopyrite
17	Ko	2013	The role of Acidithiobacillus ferrooxidans and Acidithiobacillus thiooxidans in arsenic bioleaching from soil
18	Willems	1992	Transfer of Several Phytopathogenic Pseudomonas Species to Acidovorax as Acidovorax avenae subsp. avenae subsp. nov., comb. nov., Acidovorax avenae subsp. citrulli, Acidovorax avenae subsp. cattleyae, and Acidovorax konjaci
19	Guettler	1999	Actinobacillus succinogenes sp. nov., a novel succinic-acid producing strain from the bovine rumen
20	Bruhlmann	1994	Pectinolytic Enzymes from Actinomycetes for the Degumming of Ramie Bast Fibers
21	Bruns	2003	Aeromicrobium marinum sp. nov., an abundant pelagic bacterium isolated from the German Wadden Sea
22	Abbott	2002	The Genus Aeromonas: Biochemical Characteristics, Atypical Reactions, and Phenotypic Identification Schemes
23	Siebers	2005	Unusual pathways and enzymes of central carbohydrate metabolism in archaea
24	Stacy	2014	Bacterial fight-and-flight responses enhance virulence in a polymicrobial infection
25	Todar	-	Todars Online Textbook of Bacteriology
26	Dagorn	2013	Effect of GABA, a Bacterial Metabolite, on Pseudomonas fluorescens Surface Properties and Cytotoxicity
27	Derrien	2004	Akkermansia muciniphila gen. nov., sp. nov., a human intestinal mucin-degrading bacteria
28	Derrien	2010	Mucin-bacterial interactions in the human oral cavity and digestive tract
29	Killer	2011	Fermentation of mucin by bifidobacteria from rectal samples of humans and rectal and intestinal samples of animals
30	Tailford	2015	Mucin glycan foraging in the human gut microbiome
31	Ze	2013	Some are more equal than others: the role of "keystone" species in the degradation of recalcitrant substrates
32	Rautio	2003	Reclassification of Bacteroides putredinis (Weinberg et al., 1937) in a New Genus Alistipes gen. nov., as Alistipes putredinis comb. nov., and Description of Alistipes finegoldii sp. nov., from Human Sources
33	Song	2006	Alistipes onderdonkii sp. nov. and Alistipes shahii sp. nov., of human origin
34	Sieber	2012	Genomic insights into syntrophy: The paradigm for anaerobic metabolic cooperation
35	Ezaki	2001	Proposal of the genera Anaerococcus gen. nov., Peptoniphilus gen. nov. and Gallicola gen. nov. for members of the genus Peptostreptococcus
36	Falony	2006	Cross-feeding between bifidobacterium longum BB536 and acetate-convertin, butyrate-producing colon bacteria during growth on oligofructose
37	Flint	2007	Interactions and competition within the microbial community of the human colon: links between diet and health
38	Louis	2009	Diversity, metabolism and microbial ecology of butyrate-producing bacteria from large intestine
39	Macfarlane	2012	Bacteria, colonic fermentation, and gastrointestinal health
40	Pryde	2002	The microbiology of butyrate formation in the human colon
41	Sato	2008	Isolation of lactate-utilizing butyrate-producing bacteria from human feces andin vivo administration ofAnaerostipes caccae strain L2 and galacto-oligosaccharides in a rat model
42	Scott	2013	Prebiotic stimulation of human colonic butyrate-producing bacteria and bifidobacteria, in vitro
43	Belenguer	2006	Two Routes of Metabolic Cross-Feeding between Bifidobacterium adolescentis and Butyrate-Producing Anaerobes from the Human Gut
44	Belenguer	2007	Impact of pH on Lactate Formation and Utilization by Human Fecal Microbial Communities
45	Charrier	2006	A novel class of coa-transferase involved in short-chain fatty acid metabolism in butyrate-producing human colonic bacteria
46	Duncan	2004	Lactate-Utilizing Bacteria, Isolated from Human Feces, That Produce Butyrate as a Major Fermentation Product
47	Lawson	2004	Anaerotruncus colihominis gen. nov., sp. nov., from human faeces
48	Drake	2008	Old acetogens, new light
49	Haba	2000	Isolation of lipase-secreting bacteria by deploying used frying oil as selective substrate
50	Shields	2013	Efficacy of a Marine Bacterial Nuclease against Biofilm Forming Microorganisms Isolated from Chronic Rhinosinusitis
51	Willerding	2011	Lipase Activity among Bacteria Isolated from Amazonian Soils
52	Balestrazzi	2007	Nuclease-producing bacteria in soil cultivated with herbicide resistant transgenic white poplars
53	Bentley	1982	Biosynthesis of Vitamin K (Menaquinone) in Bacteria
54	LeBlanc	2011	B-group vitamin production by lactic acid bacteria - Current knowledge and potential applications
55	Leviton	1952	Microbiological Synthesis of Vitamin B12 by Propionic Acid Bacteria
56	Martens	2002	Microbial production of vitamin B12
57	Rodionov	2003	Comparative genomics of the vitamin B12 metabolism and regulation in prokaryotes
58	Degrassi	1997	Purification and Characterization of an Acetyl Xylan Esterase from Bacillus pumilus
59	Giannella	1971	Vitamin B12 uptake by intestinal microorganisms: mechanism and relevance to syndromies of intestinal bacterial growth
60	Saxena	2003	Purification strategies for microbial lipases
61	Heinken	2013	Systems-level characterization of a host-microbe metabolic symbiosis in the mammalian gut
62	Hermann	2003	Industrial production of amino acids by coryneform bacteria
63	LeBlanc	2013	Bacteria as vitamin suppliers to their host: a gut microbiota perspective
64	Meyers	1996	Lipase production by lactic acid bacteria and activity on butter oil
65	Rodionov	2009	A novel class of modular transporters for vitamins in prokaryotes
66	Shimizu	2008	Vitamins and Related Compounds: Microbial Production, in Biotechnology: Special Processes
67	Takeno	2007	Anaerobic growth and potential for amino acid production by nitrate respiration in Corynebacterium glutamicum
68	Thompson	2012	Metabolism of sugars by genetically diverse species of oral Leptotrichia
69	Berstenhorst	2009	Vitamins and Vitamin-like Compounds: Microbial Production
70	Burke	1982	Bacillus subtilis Extracellular Nuclease Production Associated with the spoOH Sporulation Locus
71	Burkholder	1942	Synthesis of vitamins by intestinal bacteria
72	Koropatkin	2012	How glycan metabolism shapes the human gut microbiota
73	Macfarlane	2005	Colonization of Mucin by Human Intestinal Bacteria and Establishment of Biofilm Communities in a Two-Stage Continuous Culture System
74	McNulty	2011	The impact of a consortium of fermented milk strains on the gut microbiome of gnotobiotic mice and monozygotic twins
75	Sonnenburg	2010	Specificity of Polysaccharide Use in Intestinal Bacteroides Species Determines Diet-Induced Microbiota Alterations
76	Cuskin	2015	Human gut Bacteroidetes can utilize yeast mannan through a selfish mechanism
77	Chassard	2010	The cellulose-degrading microbial community of the human gut varies according to the presence or absence of methanogens
78	Flint	2012	Microbial degradation of complex carbohydrates in the gut
79	Flint	2008	Polysaccharide utilization by gut bacteria: potential for new insights from genomic analysis
80	Shah	1989	Proposal To Restrict the Genus Bacteroides (Castellani and Chalmers) to Bacteroides fragilis and Closely Related Species
81	Krieg	2010	Bergey's Manual of Systematic Bacteriology, Volume 4: The Bacteroidetes, Spirochaetes, Tenericutes (Mollicutes), Acidobacteria, Fibrobacteres, Fusobacteria, Dictyoglomi, Gemmatimonadetes, Lentisphaerae, Verrucomicrobia, Chlamydiae, and Planctomycetes
82	Deguchi	1992	Nutritional Requirements in Multiple Auxotrophic Lactic Acid Bacteria: Genetic Lesions Affecting Amino Acid Biosynthetic Pathways in Lactococcus lactis, Enterococcus faecium, and Pediococcus acidilactici
83	Nishiyama	2009	Bacteroides graminisolvens sp. nov., a xylanolytic anaerobe isolated from a methanogenic reactor treating cattle waste
84	Holdeman	1974	New Genus, Coprococcus, Twelve New Species, and Emended Descriptions of Four Previously Described Species of Bacteria from Human Feces
85	Macy	1979	The Biology of Gastrointestinal Bacteroides
86	Salyers	1977	Fermentation of Mucin and Plant Polysaccharides by Strains of Bacteroides from the Human Colon
87	Dodd	2010	Transcriptomic Analyses of Xylan Degradation by Prevotella bryantii and Insights into Energy Acquisition by Xylanolytic Bacteroidetes
88	Macfarlane	1992	Synthesis and Release of Proteases by Bacteroides fragilis
89	Macfarlane	1991	Formation of glycoprotein degrading enzymes by Bacteroides fragilis
90	Macfarlane	1986	Protein Degradation by Human Intestinal Bacteria
91	McBain	1998	Ecological and physiological studies on large intestinal bacteria in relation to production of hydrolytic and reductive enzymes involved in formation of genotoxic metabolites
92	Ridlon	2005	Bile salt biotransformations by human intestinal bacteria
93	Schink	1987	Pathway of propionate formation from ethanol in Pelobacter propionicus
94	Fukiya	2009	Conversion of cholic acid and chenodeoxycholic acid into their 7-oxo derivatives by Bacteroides intestinalis AM-1 isolated from human feces
95	Hatamoto	2014	Bacteroides luti sp. nov., an anaerobic, cellulolytic and xylanolytic bacterium isolated from methanogenic sludge
96	Martens	2011	Recognition and Degradation of Plant Cell Wall Polysaccharides by Two Human Gut Symbionts
97	Goodman	2009	Identifying Genetic Determinants Needed to Establish a Human Gut Symbiont in Its Habitat
98	Smith	1996	Studies on Amine Production in the Human Colon: Enumeration of Amine forming Bacteria and Physiological Effects of Carbohydrate and pH
99	Rakoff-Nahoum	2014	An Ecological Network of Polysaccharide Utilization among Human Intestinal Symbionts
100	Jenkins	1982	Differences in susceptibilities of species of the Bacteroides fragilis group to several beta-lactam antibiotics: indole production as an indicator of resistance.
101	Endo	2012	Comparison of Fructooligosaccharide Utilization by Lactobacillus and Bacteroides Species
102	Cato	1976	Reinstatement of Species Rank for Bacteroides fragilis, B. ovatus, B. distasonis, B. thetaiotaomicron, and B. vulgatus: Designation of Neotype Strains for Bacteroides fragilis (Veillon and Zuber) Castellani and Chalmers and Bacteroides thetaiotaomicron (Distaso) Castellani and Chalmers
103	Macfarlane	2003	Regulation of short-chain fatty acid production
104	Cooke	2006	Newly identified vitamin K-producing bacteria isolated from the neonatal faecal flora
105	Johnson	1986	Bacteroides caccae sp. nov., Bacteroides merdae sp. nov., and Bacteroides stercoris sp. nov. Isolated from Human Feces
106	Mahowald	2009	Characterizing a model human gut microbiota composed of members of its two dominant bacterial phyla
107	Musso	2011	Interaction between gut microbiota and host metabolism predisposing to obesity and diabetes
108	Payne	2012	Gut microbial adaptation to dietary consumption of fructose, artificial sweeteners and sugar alcohols
109	Rey	2013	Metabolic niche of a prominent sufate-reducing human gut bacterium
110	Rey	2010	Dissecting the in vivo metabolic potential of two human gut acetogens
111	Samuel	2006	A humanized gnotobiotic mouse model of host-archaeal-bacterial mutualism
112	Samuel	2007	Genomic and metabolic adaptations of Methanobrevibacter smithii to the human gut
113	Shoaie	2013	Understanding the interactions between bacteria in the human gut through metabolic modeling
114	Smith	1998	Enumeration of amino acid fermenting bacteria in the human large intestine: effects of pH and starch on peptide metabolsim and dissimilation of amino acids
115	Sonnenburg	2006	A hybrid two-component system protein of a prominent human gut symbiont couples glycan sensing in vivo to carbohydrate metabolism
116	Sonnenburg	2005	Glycan Foraging in Vivo by an Intestine-Adapted Bacterial Symbiont
117	Ze	2012	Ruminococcus bromii is a keystone species for the degradation of resistant starch in the human colon
118	Backhed	2005	Host-bacterial mutualism in the human intestine
119	Blaut	2013	Ecology and physiology of the intestinal tract
120	Chassard	2008	Bacteroides xylanisolvens sp. nov., a xylan-degrading bacterium isolated from human faeces
121	Degnan	2014	Human Gut Microbes Use Multiple Transporters to Distinguish Vitamin B12 Analogs and Compete in the Gut
122	Fischbach	2011	Eating for two: How metabolism establishes interspecies interactions in the gut
123	Gibson	2004	Dietary modulation of the human colonic microbiota: updating the concept of prebiotics
124	Kayahara	1994	Δ22-β-Muricholic acid in monoassociated rats and conventional ratsacid in monoassociated rats and conventional rats
125	Conly	1993	The absorption and bioactivity of bacterially synthesized menaquinones
126	Pokusaeva	2011	Cellodextrin Utilization by Bifidobacterium breve UCC2003
127	Pompei	2007	Folate production by bifidobacteria as a potential probiotic property p-aminobenzoic acid?
128	Ramirez-Farias	2009	Effect of inulin on the human gut microbiota: stimulation of Bifidobacterium adolescentis and Faecalibacterium prausnitzii
129	Rossi	2011	Folate production by probiotic bacteria
130	Rossi	2005	Fermentation of Fructooligosaccharides and Inulin by Bifidobacteria: a Comparative Study of Pure and Fecal Cultures
131	Rossi	2010	Bifidobacteria: Genomics and Molecular Aspects (Chapter 6. Probiotic properties of bifidobacteria)
132	Salyers	1977	Fermentation of Mucins and Plant Polysaccharides by Anaerobic Bacteria from the Human Colon
133	Tanaka	1999	Screening of lactic acid bacteria for bile salt hydrolase activity
134	Vernazza	2005	Carbohydrate preference, acid tolerance and bile tolerance in five strains of Bifidobacterium
135	Aachary	2011	Xylooligosaccharides (XOS) as an Emerging Prebiotic: Microbial Synthesis, Utilization, Structural Characterization, Bioactive Properties, and Applications
136	Barrett	2012	gamma-Aminobutyric acid production by culturable bacteria from the human intestine
137	Crociani	1994	Degradation of complex carbohydrates by Bifidobacterium spp.
138	Hojo	2007	Reduction of vitamin K concentration by salivary Bifidobacterium strains and their possible nutritional competition with Porphyromonas gingivalis
139	Kaplan	2000	Fermentation of Fructooligosaccharides by Lactic Acid Bacteria and Bifidobacteria
140	Wilson	2004	Microbial inhabitants of humans (Table 9.9)
141	Corfield	1992	Mucin degradation in the human colon: production of sialidase, sialate O-acetylesterase, N-acetylneuraminate lyase, arylesterase, and glycosulfatase activities by strains of fecal bacteria
142	Hoskins	1981	Mucin degradation in human colon ecosystems
143	Katayama	2005	Novel bifidobacterial glycosidases acting on sugar chains of mucin glycoproteins
144	Peterson	1945	Relation of bacteria to vitamins and other growth factors
145	Ruas-Madiedo	2008	Mucin degradation by bifidobacterium strains isolated from the human intestinal microbiota
146	Menard	2004	Lactic acid bacteria secrete metabolites retaining anti-inflammatory properties after intestinal transport
147	Pokusaeva	2010	Ribose utilization by the human commensal Bifidobacterium breve UCC2003
148	Scott	2011	Substrate-driven gene expression in Roseburia inulinivorans: Importance of inducible enzymes in the utilization of inulin
149	Degnan	1995	Arabinogalactan utilization in continuous cultures of bifidobacterium longum: Effect of co-culture with bacteroides thetaiotamicrobon
150	Kamra	2005	Rumen microbial ecosystem
151	Sela	2008	The genome sequence of Bifidobacterium longum subsp. infantis reveals adaptations for milk utilization within the infant microbiome
152	Vitali	2010	Impact of a synbiotic food on the gut microbial ecology and metabolic profiles
153	Wang	2008	Effects of the in vitro fermentation of oligofructose and inulin by bacteria growing in the human large intestine
154	Barcenilla	2000	Phylogenetic Relationships of Butyrate-Producing Bacteria from the Human Gut
155	Li	2008	Symbiotic gut microbes modulate human metabolic phenotypes
156	David	2013	Diet rapidly and reproducibly alters the human gut microbiome
157	Devkota	2012	Dietary-fat-induced taurocholic acid promotes pathobiont expansion and colitis in Il10-/- mice
158	Laue	1997	Taurine reduction in anaerobic respiration of Bilophila wadsworthia RZATAU
159	Nava	2012	Abundance and diversity of mucosa-associated hydrogenotrophic microbes in the healthy human
160	Silva	2008	Hydrogen as an energy source for the human pathogen Bilophila wadsworthia
161	Liu	2008	Reclassification of Clostridium coccoides, Ruminococcus hansenii, Ruminococcus hydrogenotrophicus, Ruminococcus luti, Ruminococcus productus and Ruminococcus schinkii as Blautia coccoides gen. nov., comb. nov., Blautia hansenii comb. nov., Blautia hydrogenotrophica comb. nov., Blautia luti comb. nov., Blautia producta comb. nov., Blautia schinkii comb. nov. and description of Blautia wexlerae sp. nov., isolated from human faeces
162	Nakamura	2010	Mechanisms of microbial hydrogen disposal in the human colon and implications for health and disease
163	Bernalier	1996	Ruminococcus hydrogenotrophicus sp. nov., a new H2/CO2-utilizing acetogenic bacterium isolated from human feces
164	Chassard	2006	H2 and acetate transfers during xylan fermentation between a butyrate-producing xylanolytic species and hydrogenotrophic microorganisms from the human gut
165	Jorda	1982	Transfer of Rhizobium japonicum Buchanan 1980 to Bradyrhizobium gen. nov., a Genus of Slow-Growing, Root Nodule Bacteria from Leguminous Plants
166	Douglas	1998	Nutritional Interactions in Insect-Microbial Symbioses: Aphids and Their Symbiotic Bacteria Buchnera
167	Perez-Brocal	2006	A small microbial genome: the end of a long symbiotic relationship?
168	Park	2007	Characterization of an Extracellular Lipase in Burkholderia sp. HY-10 Isolated from a Longicorn Beetle
169	Jaeger	1994	Bacterial lipases
170	Chistoserdova	2009	The expanding world of methylotrophic metabolism
171	Moon	2008	Reclassification of Clostridium proteoclasticum as Butyrivibrio proteoclasticus comb. nov., a butyrate-producing ruminal bacterium
172	Russell	1985	Fermentation of Cellodextrins by Cellulolytic and Noncellulolytic Rumen Bacteria
173	Wallace	1997	Metabolism of nitrogen-contraining compounds
174	Wallace	1985	The Role of Different Species of Bacteria in the Hydrolysis of Protein in the Rumen
175	Wallace	1985	Synergism between different species of proteolytic rumen bacteria
176	Cotta	1986	Proteolytic Activity of the Ruminal Bacterium Butyrivibrio fibrisolvens
177	Dehority	1991	Effects of microbial synergism on fibre digestion in the rumen
178	Duncan	2002	Acetate utilization and butyryl coenzyme A(CoA):acetate-CoA transferase in butyrate-producing bacteria from human large intestine
179	Harfoot	1997	Lipid metabolism in the rumen
180	Martin	1994	Nutrient transport by ruminal bacteria
181	Kelly	2010	The Glycobiome of the Rumen Bacterium Butyrivibrio proteoclasticus B316T Highlights Adaptation to a Polysaccharide-Rich Environment
182	Brunecky	2013	Revealing natures cellulase diversity: the digestion mechanism of caldicellulosirutpor bescii
183	Iino	2008	Calditerrivibrio nitroreducens gen. nov., sp. nov., a thermophilic, nitrate-reducing bacterium isolated from a terrestrial hot spring in Japan
184	Voordeckers	2005	Caminibacter mediatlanticus sp. nov., a thermophilic, chemolithoautotrophic, nitrate-ammonifying bacterium isolated from a deep-sea hydrothermal vent on the Mid-Atlantic Ridge
185	Wexler	1996	Sutterella wadsworthensis gen. nov., sp. nov., Bile-Resistant Microaerophilic CampyZobacter gracilis-Like Clinical Isolates
186	Hanfrey	2011	Alternative spermidine biosynthetic route is critical for growth of Campylobacter jejuni and is the dominant polyamine pathway in human gut microbiota
187	McCutcheon	2007	Parallel genomic evolution and metabolic interdependence in an ancient symbiosis
188	Wu	2006	Metabolic Complementarity and Genomics of the Dual Bacterial Symbiosis of Sharpshooters
189	Kageyama	2000	Catenibacterium mitsuokai gen. nov., sp. nov., a Gram-positive anaerobic bacterium isolated from human faeces
190	Miller	2011	Complete genome sequence of the cellulose-degrading bacterium cellulosilyticum lentocellum
191	Garrity	2005	Bergey's Manual of Systematic Bacteriology, Volume 2 : The Proteobacteria
192	Mead	1971	The Amino Acid-fermenting Clostridia
193	Milne	2011	Metabolic network reconstruction and genome-scale model of butanol-producing strain clostridium beijerinckii ncimb 8052
194	Salimi	2010	Genome-scale metabolic modeling of a clostridial co-culture for consolidated bioprocessing
195	Lee	2005	Evidence for the presence of an alternative glucose transport system in clostridium beijerinckii ncimb 8052 and the solvent-hyperproducing mutant ba101
196	Chen	1999	Effect of acetate on molecular and physiological aspects of clostridium beijerinckii ncimb 8052 solvent production and strain degeneration
197	McSweeney	1999	Isolation and Characterization of Proteolytic Ruminal Bacteria from Sheep and Goats Fed the Tannin-Containing Shrub Legume Calliandra calothyrsus
198	Elsden	1979	Amino acid utilization patterns in clostridial taxonomy
199	Nakanishi	2003	Effects of high amylose maize starch and Clostridium butyricum on metabolism in colonic microbiota and formation of azoxymethane-induced aberrant crypt foci in the rat colon
200	Seedorf	2008	The genome of Clostridium kluyveri, a strict anaerobe with unique metabolic features
201	Aklujkar	2012	The genome of Pelobacter carbinolicus reveals surprising metabolic capabilities and physiological features
202	Payot	1998	Metabolism of cellobiose by Clostridium cellulolyticum growing in continuous culture: evidence for decreased nadh reoxidation as a factor limiting growth
203	McGarr	2005	Diet, anaerobic bacterial metabolism, and colon cancer
204	Stams	1994	Metabolic interactions between anaerobic bacteria in methanogenic environments
205	Stams	2003	Metabolic interactions between methanogenic consortia and anaerobic respiring bacteria
206	Holmstrom	2004	Subdoligranulum variabile gen. nov., sp. nov. from human feces
207	Eudes	2008	Identification of genes encoding the folate and thiamine binding membrane proteins in firmicutes
208	Macfarlane	1988	Contribution of the microflora to proteolysis in the human large intestine
209	Smith	1997	Dissimilatory amino acid metabolism in human colonic bacteria
210	Starr	2006	Role of hyaluronidase in subcutaneous spread and growth of group A streptococcus
211	Vince	1980	Ammonia production by intestinal bacteria: The effect of lactose, lactulose and glucose
212	Zukaite	2000	Acceleration of hyaluronidase production in the course of batch cultivation of Clostridium perfringens can be achieved with bacteriolytic enzymes
213	van B. Robertson	1940	Mucinase: A bacterial enzyme which hydrolyzes synovial fluid mucin and other mucins
214	Attwood	1998	Ammonia-Hyperproducing Bacteria from New Zealand Ruminants
215	Johnson	2009	Interspecies Signaling between Veillonella atypica and Streptococcus gordonii Requires the Transcription Factor CcpA
216	Taras	2002	Reclassification of Eubacterium formicigenerans Holdeman and Moore 1974 as Dorea formicigenerans gen. nov., comb. nov., and description of Dorea longicatena sp. nov., isolated from human faeces
217	Kageyama	2000	Emendation of genus Collinsella and proposal of Collinsella stercoris sp. nov. and Collinsella intestinalis sp. nov.
218	Newsholme	1994	Quantitative aspects of glucose and glutamine metabolism by intestinal cells
219	Roediger	1997	Human colonocyte detoxification
220	Thiele	2013	A community-driven global reconstruction of human metabolism
221	Turnberg	1970	Electrolyte absorption from the colon
222	Yu	2010	Bestrophin-2 mediates bicarbonate transport by goblet cells in mouse colon
223	Bergen	2009	Intestinal Nitrogen Recycling and Utilization in Health and Disease
224	Chen	2010	Microbial and Bioconversion Production of D-xylitol and Its Detection and Application
225	Conly	1994	The contribution of vitamin K2(menaquinones) produced by the intestinal microflora to human nutritional requirements for vitamin K
226	Ganong	1991	Review of medical physiology. Section V. 25. Digestion & absorption
227	Hughes	2011	Protein Degradation in the Large Intestine: Relevance to Colorectal Cancer
228	Suen	2011	The Complete Genome Sequence of Fibrobacter succinogenes S85 Reveals a Cellulolytic and Metabolic Specialist
229	Russell	1981	Degradation of Protein by Mixed Cultures of Rumen Bacteria: Identification of Streptococcus Bovis as an Actively Proteolytic rumen bacterium
230	Holland	2006	Development of a defined medium supporting rapid growth for Deinococcus radiodurans and analysis of metabolic capacities
231	Myhr	2000	Denitrovibrio acetiphilus, a novel genus and species of dissimilatory nitrate-reducing bacterium isolated from an oil reservoir model column
232	Schmidt	1995	Interspecies Electron Transfer during Propionate and Butyrate Degradation in Mesophilic, Granular Sludge
233	Imachi	2002	Pelotomaculum thermopropionicum gen. nov., sp. nov., an anaerobic, thermophilic, syntrophic propionate-oxidizing bacterium
234	Cord-Ruwisch	1998	Growth of Geobacter sulfurreducens with Acetate in Syntrophic Cooperation with Hydrogen-Oxidizing Anaerobic Partners
235	Klitgord	2010	Environments that induce synthetic microbial ecosystems
236	Stolyar	2007	Metabolic modeling of a mutualistic microbial community
237	Kosaka	2008	The genome of Pelotomaculum thermopropionicum reveals niche-associated evolution in anaerobic microbiota
238	Lovley	1993	Geobacter metallireducens gen. nov. sp. nov., a microorganism capable of coupling the complete oxidation of organic compounds to the reduction of iron and other metals
239	Downes	2003	Dialister invisus sp. nov., isolated from the human oral cavity
240	Pircher	2007	Formation of cadaverine, histamine, putrescine and tyramine by bacteria isolated from meat, fermented sausages and cheeses
241	Schleifer	1984	Transfer of Streptococcus faecalis and Streptococcus faecium to the Genus Enterococcus norn. rev. as Enterococcus faecalis comb. nov. and Enterococcus faecium comb. nov.
242	Vos	2009	Bergey's Manual of Systematic Bacteriology, Volume 3: The Firmicutes
243	Deibel	1964	Pyruvate fermentation by Streptococcus faecalis
244	Delk	1975	Biosynthesis of Ribosylthymine in the TransferRNA of Streptococcus faecalis: A Folate-Dependent Methylation Not Involving S-Adenosylmethionine
245	Bos	2013	Volatile Metabolites of Pathogens: A Systematic Review
246	Mulligan	1977	Transport and metabolism of vitamin B6 in lactic acid bacteria
247	Ryu	2001	Characteristics and Glycerol Metabolism of Fumarate-Reducing Enterococcus faecalis RKY1
248	Wang	2009	Bio-hydrogen production from cellulose by sequential co-culture of cellulosic hydrogen bacteria of Enterococcus gallinarum G1 and Ethanoigenens harbinense B49
249	Ryan	2004	Sherris Medical Microbiology (4th ed.)
250	Tang	2005	Xanthomonas campestris pv. campestris possesses a single gluconeogenic pathway that is required for virulence
251	Sawers	2005	Formate and its role in hydrogen production in Escherichia coli
252	Clark	1989	The fermentation pathways of Escherichia coli.
253	Peekhaus	1998	What’s for Dinner?: Entner-Doudoroff Metabolism in Escherichia coli
254	Jones	2011	Anaerobic Respiration of Escherichia coli in the Mouse Intestine
255	Van der Werf	1997	Environmental and physiological factors affecting the succinate product ratio during carbohydrate fermentation by Actinobacillus sp. 130Z
256	Sirko	1995	Sulfate and Thiosulfate Transport in Escherichia coli K-12: Evidence for a Functional Overlapping of Sulfate-and Thiosulfate-Binding Proteins
257	Walker	2011	Dominant and diet-responsive groups of bacteria within the human colonic microbiota
258	Duncan	2002	Roseburia intestinalis sp. nov., a novel saccharolytic, butyrate-producing bacterium from human faeces
259	Duncan	2003	Effects of Alternative Dietary Substrates on Competition between Human Colonic Bacteria in an Anaerobic Fermentor System
260	Chen	1989	More Monensin-Sensitive, Ammonia-Producing Bacteria from the Rumen
261	Venkataraman	2014	Metabolite transfer with the fermentation product 2,3-butanediol enhances virulence by Pseudomonas aeruginosa
262	McBride	2009	Novel Features of the Polysaccharide-Digesting Gliding Bacterium Flavobacterium johnsoniae as Revealed by Genome Sequence Analysis
263	Chen	1988	Fermentation of peptides and amino acids by monensin-sensitive ruminal peptostreptococcus
264	Loesche	1968	Amino acid fermentation by Fusobacterium nucleatum
265	Yang	2013	Isolation and Characterization of Novel Denitrifying Bacterium Geobacillus sp. SG-01 Strain from Wood Chips Composted with Swine Manure
266	Heider	1999	Anaerobic bacterial metabolism of hydrocarbons
267	Nagarajan	2013	Characterization and modeling of interspecies electron transfer mechanisms and microbial community dynamics of a syntrophic association
268	Schleinitz	2009	Phenol Degradation in the Strictly Anaerobic Iron-Reducing Bacterium Geobacter metallireducens GS-15
269	Summers	2010	Direct exchange of electrons within aggregates of an evolved syntrophic coculture of anaerobe bacteria
270	Manzoni	2001	Biotransformation of D-galactitol to tagatose by acetic acid bacteria
271	Sato	2011	Novel metabolic pathways in archaea
272	Severina	1991	Glucose transport into the extremely halophilic archaebacteria
273	Albers	2004	Insights into ABC transport in archaea
274	Deplancke	2001	Microbial modulation of innate defense: goblet cells and the intestinal mucus layer
275	Gibson	1996	Fermentation of non-digestible oligosaccharides by human colonic bacteria
276	Marshall	1990	Urea protects Helicobacter (Campylobacter) pylori from the bactericidal effect of acid
277	McNulty	2013	Mechanisms of molecular transport through the urea channel of Helicobacter pylori
278	Wilems	1997	Phenotypic and Phylogenetic Characterization of some Eubacterium-like isolates containing a novel type B wall murein from human feces: description of Holdemania filiformis gen. nov., sp. nov.
279	Urakami	1995	Characterization and Description of Hyphomicrobium denitrificans sp. nov.
280	Wrede	2012	Archaea in symbioses
281	Oppenberg	1990	Anaerobic degradation of 1,3-propanediol by sulfate-reducing and by fermenting bacteria
282	Snell	1976	Transfer of Some Saccharolytic Moraxella Species to Kingella Henriksen and Bovre 1976, with Descriptions of Kingella indologenes sp. nov. and Kingella denitrificans sp. nov.
283	Ahrens	1997	Kinetic, Dynamic, and Pathway Studies of Glycerol Metabolism by Klebsiella pneumoniae in Anaerobic Continuous Culture: III. Enzymes and Fluxes of Glycerol Dissimilation and 1,3-Propanediol Formation
284	Hung	2011	Facultative methylotrophs from the human oral cavity and methylotrophy in strains of Gordonia, Leifsonia, and Microbacterium
285	Keuth	1994	Vitamin B12 production by Citrobacter freundii or Klebsiella pneumoniae during tempeh fermentation and proof of enterotoxin absence by PCR
286	Liao	2012	An Experimentally Validated Genome-Scale Metabolic Reconstruction of Klebsiella pneumoniae MGH 78578, iYL1228
287	Begley	2005	The interaction between bacteria and bile
288	Rao	1984	Biosynthesis and Utilization of Folic Acid and Vitamin B12 by Lactic Cultures in Skim Milk
289	Rooj	2010	Metabolites produced by probiotic lactobacilli rapidly increase glucose uptake by caco-2 cells
290	Almståhl	2013	Fermentation of sugars and sugar alcohols by plaque Lactobacillus strains
291	Veiga	1992	Sugar-Glycerol Cofermentations in Lactobacilli: the Fate of Lactate
292	González-Pajuelo	2006	Microbial Conversion of Glycerol to 1,3-Propanediol: Physiological Comparison of a Natural Producer, Clostridium butyricum VPI 3266, and an Engineered Strain, Clostridium acetobutylicum DG1(pSPD5)
293	Hugenschmidt	2010	Screening of a natural biodiversity of lactic and propionic acid bacteria for folate and vitamin B12 production in supplemented whey permeate
294	Oude Elferink	2001	Anaerobic Conversion of Lactic Acid to Acetic Acid and 1,2-Propanediol by Lactobacillus buchneri
295	Cselovsky	1992	Production of formate, acetate, and succinate by anaerobic fermentation of Lactobacillus pentosus in the presence of citrate
296	Henderson	1979	Mechanism of Folate Transport in Lactobacillus casei: Evidence for a Component Shared with the Thiamine and Biotin Transport Systems
297	Thompson	1987	Regulation of sugar transport and metabolism in lactic acid bacteria
298	Bertelsen	2001	Fermentation of D-Tagatose by Human Intestinal Bacteria and Dairy Lactic Acid Bacteria
299	Beshkova	1998	Production of Amino Acids by Yogurt Bacteria
300	Piveteau	1995	Interactions between lactic and propionic acid bacteria
301	Taranto	2003	Lactobacillus reuteri CRL1098 Produces Cobalamin
302	Robbins	1940	Fermentation of Sugar Acids by Bacteria
303	Wegkamp	2004	Transformation of Folate-Consuming Lactobacillus gasseri into a Folate Producer
304	Klein	1998	Taxonomy and physiology of probiotic lactic acid bacteria
305	Fujisawa	1992	Taxonomic Study of the Lactobacillus acidophilus Group, with Recognition of Lactobacillus gallinarum sp. nov. and Lactobacillus johnsonii sp. nov. and Synonymy of Lactobacillus acidophilus Group A3 (Johnson et l. 1980) with the Type Strain of Lactobacillus amylovorus (Nakamura 1981)
306	Collins	1989	Deoxyribonucleic Acid Homology Studies of Lactobacillus casei, Lactobacillus paracasei sp. nov., subsp. paracasei and subsp. tolerans, and Lactobacillus rhamnosus sp. nov., comb. nov.
307	Hedberg	2008	Sugar fermentation in probiotic bacteria – an in vitro study
308	Hugenschmidt	2011	Concurrent high production of natural folate and vitamin B12 using a co-culture process with lactobacillus plant arum SM39 and propionibacterium freudenreichii DF13
309	Lindgren	1990	Anaerobic L-lactate degradation by lactobacillus plantarum
310	Santos	2008	High-Level Folate Production in Fermented Foods by the B12 Producer Lactobacillus reuteri JCM1112
311	Talarico	1990	Utilization of Glycerol as a Hydrogen Acceptor by Lactobacillus reuteri: Purification of 1,3-Propanediol:NAD+ Oxidoreductaset
312	Zelante	2013	Tryptophan Catabolites from Microbiota Engage Aryl Hydrocarbon Receptor and Balance Mucosal Reactivity via Interleukin-22
313	Rodríguez	2012	Mannitol production by heterofermentative Lactobacillus reuteri CRL 1101 and Lactobacillus fermentum CRL 573 in free and controlled pH batch fermentations.
314	Mao	2013	Bacteroides fragilis polysaccharide A is necessary and sufficient for acute activation of intestinal sensory neurons
315	Eribe	2004	Genetic diversity of Leptotrichia and description of Leptotrichia goodfellowii sp. nov., Leptotrichia hofstadii sp. nov., Leptotrichia shahii sp. nov. and Leptotrichia wadei sp. nov
316	Kihal	2007	Carbon dioxide production by Leuconostoc mesenteroides grown in single and mixed culture with Lactococcus lactis in skimmed milk
317	Erten	1998	Metabolism of fructose as an electron acceptor by Leuconostoc mesenteroides
318	Begley	2006	Bile Salt Hydrolase Activity in Probiotics
319	Wolin	2003	Formate-Dependent Growth and Homoacetogenic Fermentation by a Bacterium from Human Feces: Description of Bryantella formatexigens gen. nov., sp. nov
320	Sakon	2008	Sutterella parvirubra sp. nov. and Megamonas funiformis sp. nov., isolated from human faeces
321	Latham	1977	Fermentation of cellulose by Ruminococcus flavefaciens in the presence and absence of Methanobacterium ruminantium
322	Le Chatelier	2013	Richness of human gut microbiome correlates with metabolic markers
323	Woese	2012	The Bacteria. A Treatise on Structure and Function. Vol. VIII Archaebacteria
324	Liu	2008	Metabolic, phylogenetic, and ecological diversity of the methanogenic archaea
325	DHaeze	2002	A hyperthermophilic methanogen sequenced
326	Fischer	2005	Structures and reaction mechanisms of riboflavin synthases of eubacterial and archaeal origin
327	Blaut	1994	Metabolism of methanogens
328	Benedict	2011	Genome-scale metabolic reconstruction and hypothesis testing in the methanogenic archaeon methanosarcina acetivorans c2a
329	Gonnerman	2013	Genomically and biochemically accurate metabolic reconstruction of methanosarcina barkeri fusaro, iMG746
330	Cadillo-Quiroz	2009	Methanosphaerula palustris gen. nov., sp. nov., a hydrogenotrophic methanogen isolated from a minerotrophic fen peatland
331	Struchtemeyer	2011	Evidence for syntrophic butyratemetabolismunder sulfatereducing conditions ina hydrocarbon-contaminated aquifer
332	Chistoserdova	2011	Modularity of methylotrophy, revisited
333	Jourand	2004	Methylobacterium nodulans sp. nov., for a group of aerobic, facultatively methylotrophic, legume root-nodule-forming and nitrogen-fixing bacteria
334	Ward	2004	Genomic Insights into Methanotrophy: The Complete Genome Sequence of Methylococcus capsulatus
335	Sakai	2007	Degradation of Glyoxylate and Glycolate with ATP Synthesis by a Thermophilic Anaerobic Bacterium, Moorella sp. Strain HUC22-1
336	Enright	1997	Moraxella (Branhamella) catarrhalis - clinical and molecular aspects of a rediscovered pathogen
337	Grant	1981	Denitrification by Strains of Neisseria, Kingella, and Chromobacterium
338	Goker	2011	Complete genome sequence of Odoribacter splanchnicus type strain (1651/6T)
339	Kuhner	1996	Generation of a proton motive force by the anaerobic oxalate-degrading bacterium Oxalobacter formigene
340	Sakamoto	2006	Reclassification of Bacteroides distasonis, Bacteroides goldsteinii and Bacteroides merdae as Parabacteroides distasonis gen. nov., comb. nov., Parabacteroides goldsteinii comb. nov. and Parabacteroides merdae comb. nov.
341	Sugawara	1991	Digestion and Fermentation by Human Intestinal Bacteria of Corn Fiber and Its Hemicellulose in Vitro
342	Sakamoto	2007	Parabacteroides johnsonii sp. nov., isolated from human faeces
343	Tanasupawat	1993	Characterization of Pediococcus pentosaceus and Pediococcus acidilactici Strains and Replacement of the Type Strain of P. acidilactici with the Proposed Neotype DSM 20284
344	Papagianni	2009	Pediocins: The bacteriocins of Pediococci. Sources, production, properties and applications
345	Schink	1984	Fermentation of 2,3-butanediol by Pelobacter carbinolicus sp. nov. and Pelobacter propionicus sp. nov., and evidence for propionate formation from C2 compounds
346	Muller	2010	Syntrophic butyrate and propionate oxidation processes: from genomes to reaction mechanisms
347	Ng	2013	Microbiota-liberated host sugars facilitate post-antibiotic expansion of enteric pathogens
348	Theriot	2014	Antibiotic-induced shifts in the mouse gut microbiome and metabolome increase susceptibility to Clostridium difficile infection
349	Holmes	2011	Understanding the role of gut microbiome-host metabolic signal disruption in health and disease
350	Accetto	2007	Studies on Prevotella nuclease using a system for the controlled expression of cloned genes in P. bryantii TC1-1
351	Hayashi	2007	Prevotella copri sp. nov. and Prevotella stercorea sp. nov., isolated from human faeces
352	Chassard	2005	Interaction between H2-producing and non-H2-producing cellulolytic bacteria from the human colon
353	Herbeck	1974	Nutritional Features of the Intestinal Anaerobe Ruminococcus bromii
354	Kabel	2011	Biochemical Characterization and Relative Expression Levels of Multiple Carbohydrate Esterases of the Xylanolytic Rumen Bacterium Prevotella ruminicola 23 Grown on an Ester-Enriched Substrate
355	Pittman	1964	Peptides and other nitrogen sources for growth of bacteroides ruminicola
356	Russell	1988	Enrichment and Isolation of a Ruminal Bacterium with a Very High Specific Activity of Ammonia Production
357	Strobel	1992	Vitamin B12-dependent propionate production by the ruminal bacterium Prevotella ruminicola 23
358	Varel	1974	Nutritional Features of Bacteroides fragilis subsp. fragilis
359	Yanagisawa	2006	Proteinase Activity of Prevotella Species Associated with Oral Purulent Infection
360	Ingram	1983	Studies of the extracellular proteolytic activity produced by Propionibacterium acnes
361	Jeter	1984	Salmonella typhimurium Synthesizes Cobalamin (Vitamin B12) De Novo Under Anaerobic Growth Conditions
362	Kosmider	2010	Propionic Acid Production by Propionibacterium freudenreichiissp. shermanii Using Crude Glycerol and Whey Lactose Industrial Wastes
363	Roth	1996	COBALAMIN (COENZYME B12): Synthesis and Biological Significance
364	Vince	1973	Ammonia production by intestinal bacteria
365	Carlier	2009	Proposal to unify Clostridium orbiscindens Winter et al. 1991 and Eubacterium plautii (Sguin 1928) Hofstad and Aasjord 1982, with description of Flavonifractor plautii gen. nov., comb. nov., and reassignment of Bacteroides capillosus to Pseudoflavonifractor capillosus gen. nov., comb. nov.
366	Chou	2008	Transcriptome analysis of agmatine and putrescine catabolism in Pseudomonas aeruginosa PAO1
367	Stuer	1986	Purification of extracellular lipase from Pseudomonas aeruginosa
368	Valentini	2011	Identification of C4-Dicarboxylate Transport Systems in Pseudomonas aeruginosa PAO1
369	Sias	1979	Isolation and analysis of mutants of Pseudomonas aeruginosa unable to assimilate nitrate.
370	Samuelsson	1988	Heat Production by the Denitrifying Bacterium Pseudomonas fluorescens and the Dissimilatory Ammonium-Producing Bacterium Pseudomonas putrefaciens during Anaerobic Growth with Nitrate as the Electron Acceptor
371	Sharma	2001	Production, purification, characterization, and applications of lipases
372	Daum	1998	Physiological and Molecular Biological Characterization of Ammonia Oxidation of the Heterotrophic Nitrifier Pseudomonas putida
373	Downes	2009	Pyramidobacter piscolens gen. nov., sp. nov., a member of the phylum Synergistetesisolated from the human oral cavity
374	Völkl	1993	Pyrobaculum aerophilum sp. nov., a Novel Nitrate-Reducing Hyperthermophilic Archaeum
375	Amo	2002	Pyrobaculum calidifontis sp. nov., a novel hyperthermophilic archaeon that grows in atmospheric air
376	Koning	2001	Cellobiose Uptake in the Hyperthermophilic Archaeon Pyrococcus furiosus Is Mediated by an Inducible, High-Afnity ABC Transporter
377	Hemachander	2001	Whole cell immobilization of Ralstonia pickettii for lipase production
378	Reichardt	2014	Phylogenetic distribution of three pathways for propionate production within the human gut microbiota
379	Doi	1991	Enhancement of Denitrifying Activity in Cells of Roseobacter denitrificans Grown Aerobically in the Light
380	Gold	2007	Global view of the clostridium thermocellum cellulosome revealed by quantitative proteomic analysis
381	Ng	1982	Differential metabolism of cellobiose and glucose by clostridum thermocellum and clostridium thermohydrosulfuricum
382	Rincon	2005	Unconventional Mode of Attachment of the Ruminococcus flavefaciens Cellulosome to the Cell Surface
383	Sparling	2006	Formate synthesis by clostridium thermocellum
384	Bryant	1960	Studies on the Nitrogen Requirements of Some Ruminal Cellulolytic Bacteria
385	Louis	2007	Understanding the effects of diet on bacterial metabolism in the large intestine
386	Shi	1997	Formation of formate and hydrogen, and flux of reducing equivalents and carbon in Ruminococcus flavefaciens FD-1
387	Allison	1962	Metabolic function of branched-chain volatile fatty acids, growth factors for ruminococci. II. Biosynthesis of higher branched-chain fatty acids and aldehydes
388	Brettar	2002	Shewanella denitrificans sp. nov., a vigorously denitrifying bacterium isolated from the oxic–anoxic interface of the Gotland Deep in the central Baltic Sea
389	Sadovski	1969	Extracellular Nuclease Activity of Fish Spoilage Bacteria, Fish Pathogens, and Related Species
390	Woodward	2005	Identification and characterization of Shigella boydii 20 serovar nov., a new and emerging Shigella serotype
391	Snyder	2010	Nutrient provisioning facilitates homeostasis between tsetse fly (Diptera: Glossinidae) symbionts
392	Arnone	1969	The extracellular nuclease of staphylococcus aureus: Structures of the native enzyme and an enzyme-inhibitor complex at 4A resolution
393	Beenken	2012	Impact of Extracellular Nuclease Production on the Biofilm Phenotype of Staphylococcus aureus under In Vitro and In Vivo Conditions
394	Willcox	2001	Streptococcus australis sp. nov., a novel oral streptococcus
395	Luppens	2008	Effect of Veillonella parvula on the antimicrobial resistance and gene expression of Streptococcus mutans grown in a dual-species biofilm
396	Loscalzo	2011	Lipid metabolism by gut microbes and atherosclerosis
397	Whiley	1990	Streptococcus parasanguis sp. nov., an atypical viridans Streptococcus from human clinical specimens
398	Benedik	1998	Serratia marcescens and its extracellular nuclease
399	Whiley	1988	Streptococcus vestibularis sp. nov. from the Human Oral Cavity
400	Nisole	2006	Extracellular production of Streptomyces lividans acetyl xylan esterase A in Escherichia coli for rapid detection of activity
401	Albers	1999	Glucose Transport in the Extremely Thermoacidophilic Sulfolobus solfataricus Involves a High-Affinity Membrane-Integrated Binding Protein
402	Mukhopadhya	2011	A Comprehensive Evaluation of Colonic Mucosal Isolates of Sutterella wadsworthensis from Inflammatory Bowel Disease
403	Boone	1989	Diffusion of the Interspecies Electron Carriers H2 and Formate in Methanogenic Ecosystems and Its Implications in the Measurement of Km for H2 or Formate Uptake
404	Sousa	2007	Syntrophomonas zehnderi sp. nov., an anaerobe that degrades long-chain fatty acids in co-culture with methanobacterium formicicum
405	Stieb	1985	Anaerobic oxidation of fatty acids by clostridium byrantii sp. nov., a sporeforming, obligately syntrophic bacterium
406	Wu	1994	Anaerobic degradation of normal- and branched-chain fatty acids with four or more carbons to methane by a syntrophic methanogenic triculture
407	Jackson	2002	Anaerobic microbial metabolism can proceed close to thermodynamic limits
408	Fulcinos	2005	Identification of extracellular lipases/esterases produced by Thermus thermophilus HB27: partial purification and preliminary biochemical characterisation
409	Kelly	2000	Confirmation of Thiobacillus denitrificans as a species of the genus Thiobacillus, in the β- subclass of the Proteobacteria, with strain NCIMB 9548 as the type strain
410	Baalsrud	1954	Studies on Thiobacillus denitrificans
411	Paralonov	2012	Comparative genome analysis of 19 Ureaplasma urealyticum and Ureaplasma parvum strains
412	Ng	1971	Lactate metabolism by Veillonella parvula
413	Seper	2011	Extracellular nucleases and extracellular DNA play important roles in Vibrio cholerae biofilm formation
414	Blokesch	2008	The Extracellular Nuclease Dns and Its Role in Natural Transformation of Vibrio cholerae
415	Balch	1997	Acetobacterium, a New Genus of Hydrogen-Oxidizing, Carbon Dioxide-Reducing,Anaerobic Bacteria
416	Buschhorn	1989	Production and Utilization of Ethanol by the Homoacetogen Acetobacterium woodii
417	Peters	1998	Efficiency of hydrogen utilization during unitrophic and mixotrophic growth of Acetobacterium woodii on hydrogen and lactate in the chemostat
418	Heise	1989	Sodium Dependence of Acetate Formation by the Acetogenic Bacterium Acetobacterium woodii
419	Zitomersky	2013	Characterization of Adherent Bacteroidales from Intestinal Biopsies of Children and Young Adults with Inflammatory Bowel Disease
420	Mishra	2012	Genome sequence and description of Alistipes senegalensis sp. nov.
421	Downes	2013	Description of Alloprevotella rava gen. nov., sp. nov., isolated from the human oral cavity, and reclassification of Prevotella tannerae Moore et al. 1994 as Alloprevotella tannerae gen. nov., comb. nov.
422	Baena	1999	Aminomonas paucivorans gen. nov., sp. nov., a mesophilic, anaerobic, amino-acid-utiIizing bacterium
423	Allen-Vercoe	2012	Anaerostipes hadrus comb. nov., a dominant species within the human colonic microbiota; reclassification of Eubacterium hadrum Moore et al. 1976
424	Moore	1976	Emendation of Bacteroidaceae and Butyrivibrio and Descriptions of Desulfornonas gen. nov. and Ten New Species in the Genera Desulfomonas, Butyrivibrio, Eubacterium, Clostridium, and Ruminococcus
425	Whitehead	2005	Bacteroides coprosuis sp. nov., isolated from swine-manure storage pits
426	Fenner	2005	Bacteroides massiliensis sp. nov., isolated from blood culture of a newborn
427	Song	2004	“Bacteroides nordii” sp. nov. and “Bacteroides salyersae” sp. nov. Isolated from Clinical Specimens of Human Intestinal Origin
428	Love	1986	Bacteroides tectum sp. nov. and Characteristics of Other Nonpigmented Bactevoides Isolates from Soft-Tissue Infections from Cats and Dogs
429	Benno	1983	Bacteroides pyogenes sp. nov., Bacteroides suis sp. nov., and Bacteroides helcogenes sp. nov., New Species from Abscesses and Feces of Pigs
430	Ezaki	1994	16s Ribosomal DNA Sequences of Anaerobic Cocci and Proposal of Ruminococcus hansenii comb. nov. and Ruminococcus productus comb. nov.
431	Kinyon	1979	Treponema innocens, a New Species of Intestinal Bacteria, and Emended Description of the Type Strain of Treponema hyodysenteriae Harris et al.
432	Stanton	1997	Recognition of Two New Species of Intestinal Spirochetes: Serpulina intermedia sp. nov. and Serpulina murdochii sp. nov.
433	Fardeau	2000	Thermoanaerobacter subterraneus sp. nov., a novel thermophile isolated from oilfield water
434	Mori	2009	Caldisericum exile gen. nov., sp. nov., an anaerobic, thermophilic, filamentous bacterium of a novel bacterial phylum, Caldiserica phyl. nov., originally called the candidate phylum OP5, and description of Caldisericaceae fam. nov., Caldisericales ord. nov. and Caldisericia classis nov.
435	Itoh	2003	Caldisphaera lagunensis gen. nov., sp. nov., a novel thermoacidophilic crenarchaeote isolated from a hot spring at Mt Maquiling, Philippines
436	Miroshnichenko	2003	Caldithrix abyssi gen. nov., sp. nov., a nitrate- reducing, thermophilic, anaerobic bacterium isolated from a Mid-Atlantic Ridge hydrothermal vent, represents a novel bacterial lineage
437	Ogg	2011	Caloramator mitchellensis sp. nov., a thermoanaerobe isolated from the geothermal waters of the Great Artesian Basin of Australia, and emended description of the genus Caloramator
438	Ogg	2009	Caloramator australicus sp. nov., a thermophilic, anaerobic bacterium from the Great Artesian Basin of Australia
439	Vandamme	2010	Reclassification of Bacteroides ureolyticus as Campylobacter ureolyticus comb. nov., and emended description of the genus Campylobacter
440	Askew	2009	Transcriptional Regulation of Carbohydrate Metabolism in the human pathogen candida albicans
441	Williamson	1986	Biotypes of Candida albicans using the API 20C system
442	Sullivan	1995	Candida dubliniensis sp. nov.: phenotypic and molecular characterization of a novel species associated with oral candidosis in HIV-infected individuals
443	Wong	1993	D-Arabitol Metabolism in Candida albicans: Studies of the Biosynthetic Pathway and the Gene That Encodes NAD-Dependent D-Arabitol Dehydrogenase
444	Granstrom	2002	Metabolic flux analysis of candida tropicalis growing on xylose in an Oxygen-limited chemostat
445	Rehman	2010	Cadmium biosorption by yeats, Candida tropical is CBL-1, isolated from industrial wastewater
446	Sulman	2013	Isolation and Characterization of Cellulose Degrading Candida tropicalis W2 from Environmental Samples
447	West	2009	Xylitol production by Candida species grown on a grass hydrolysate
448	Lohmeier-Vogel	1989	31P Nuclear Magnetic Resonance Study of the Effect of Azide on Xylose Fermentation by Candida tropicalis
449	Jiang	2007	Biodegradation of phenol and 4-chlorophenol by the yeast Candida tropicalis
450	Sudha	2010	Comparative study for the production, characterisation and antimicrobial studies of Sophorolipids using Candida tropicalis
451	Nakamura	1968	Transglucosyl-Amylase of Candida tropicalis
452	Brenner	1989	Capnocytophaga canimorsussp.nov.(FormerlyCDC GroupDF-2), a Cause of Septicemia following Dog Bite, and C. cynodegmi sp. nov., a Cause of Localized Wound Infection following Dog Bite
453	Lawson	2006	Catellicoccus marimammalium gen. nov., sp. nov., a novel Gram-positive, catalase-negative, coccus- shaped bacterium from porpoise and grey seal
454	Finegold	2003	Cetobacterium somerae sp. nov. from Human Feces and Emended Description of the Genus Cetobacterium
455	Jung	2010	Clostridium arbusti sp. nov., an anaerobic bacterium isolated from pear orchard soil
456	Abrini	1994	Clostridium autoethanogenum, sp. nov., an anaerobic bacterium that produces ethanol from carbon monoxide
457	Chamkha	2001	Isolation of Clostridium bifermentans from Oil Mill Wastewaters Converting Cinnamic Acid to 3-phenylpropionic Acid and Emendation of the Species
458	Dai	2011	Amino acid metabolism in intestinal bacteria: links between gut ecology and host health
459	Hauschild	1974	Clostridium celatum sp.nov., Isolated from Normal Human Feces
460	Warren	2006	Clostridium aldenense sp. nov. and Clostridium citroniae sp. nov. Isolated from Human Clinical Infections
461	Kaneuchi	1976	Taxonomic Study of Bacteroides dostridiiformis subsp. clostridiiformis (Burri and Ankersmit) Holdeman and Moore and of Related Organisms: Proposal of Clostridium clostridiiformis (Burri and Ankersmit) comb. nov. and Clostridium symbiosum (Stevens) comb. nov.
462	Greetham	2003	Clostridium colicanis sp. nov., from canine faeces
463	Smith	1962	Clostridium innocuum, sp. n., a spore-forming anaerobe isolated from human infections
464	Dabrock	1992	Parameters Affecting Solvent Production by Clostridium pasteurianum
465	Keis	2001	Emended descriptions of Clostridium acetobutylicum and Clostridium beijerinckii, and descriptions of Clostridium saccharoperbutylacetonicum sp. nov. and Clostridium saccharobutylicum sp. nov.
466	Partansky	1935	Anaerobic Bacteria Capable of the Fermentation of Sulfite Waste Liquor
467	Madden	1983	Isolation and Characterization of Clostridium stercorarium sp. nov., Cellulolytic Thermophile
468	Fardeau	2001	Transfer of Thermobacteroides leptospartum and Clostridium thermolacticum as Clostridium stercorarium subsp. leptospartum subsp. nov., comb. nov. and C. stercorarium subsp. thermolacticum subsp. nov., comb. nov.
469	Hethener	1992	Clostridium termitidis sp. nov., a Cellulolytic Bacterium from the Gut of the Wood-feeding Termite, Nasutitermes lujae
470	Jonsson	1990	Enumeration and Confirmation of Clostridium tyrobutyricum in Silages Using Neutral Red, D-Cycloserine, and Lactate Dehydrogenase Activity
471	Kunzelmann	2002	Electrolyte Transport in the Mammalian Colon: Mechanisms and Implications for Disease
472	Rabus	1993	Complete Oxidation of Toluene under Strictly Anoxic ConditionsbyaNew Sulfate-ReducingBacterium
473	Trinkerl	1990	Desulfovibrio termitidis sp. nov., a Carbohydrate-Degrading Sulfate-Reducing Bacterium from the Hindgut of a Termite
474	Sorokin	2008	Dethiobacter alkaliphilus gen. nov. sp. nov., and Desulfurivibrio alkaliphilus gen. nov. sp. nov.: two novel representatives of reductive sulfur cycle from soda lakes.
475	L'Haridon	1998	Desulfurobacterium thermolithotrophum gen. nov., sp. nov., a novel autotrophic, sulphur-reducing bacterium isolated from a deep-sea hydrothermal vent
476	Hofstad	2000	Dysgonomonas gen. nov. to accommodate Dysgonomonas gadei sp. nov., an organism isolated from a human gall bladder, and Dysgonomonas capnocytophagoides (formerly CDC group DF-3)
477	Lawson	2002	Dysgonomonas mossii sp. nov., from Human Sources*
478	Doran	1978	Eimeria tenella: vitamin requirements for development in primary cultures of chicken kidney cells.
479	Smith	1986	Monosaccharide Transport by Eimeria tenella Sporozoites
480	Kampfer	2011	Elizabethkingia anophelis sp. nov., isolated from the midgut of the mosquito Anopheles gambiae
481	Kim	2005	Transfer of Chryseobacterium meningosepticum and Chryseobacterium miricola to Elizabethkingia gen. nov. as Elizabethkingia meningoseptica comb. nov. and Elizabethkingia miricola comb. nov.
482	Holmes	1982	Flavobacteriurn breve sp. nov., norn. rev.
483	Saha	2006	Emticicia oligotrophica gen. nov., sp. nov., a new member of the family ‘Flexibacteraceae’, phylum Bacteroidetes
484	Mda	2006	Enterococcus caccae sp. nov., isolated from human stools
485	Svec	2001	Enterococcus haemoperoxidus sp. nov. and Enterococcus moraviensis sp. nov., isolated from water
486	Law-Brown	2003	Enterococcus phoeniculicola sp. nov., a novel member of the enterococci isolated from the uropygial gland of the Red-billed Woodhoopoe, Phoeniculus purpureus
487	Vancanneyt	2001	Enterococcus villorum sp. nov., an enteroadherent bacterium associated with diarrhoea in piglets
488	Holdeman	1971	Clostridium ramosum (Vuillemin)comb.nov.:Emended Description and Proposed-NeotypeStrain
489	Holdeman	1980	Descriptions of Eubacterium timidum sp. nov., Eubacterium brachy sp. nov., and Eubacterium nodatum sp. nov. Isolated from Human Periodontitis
490	Margaret	1986	Eubacterium yurii subsp. yurii sp. nov. and Eubacterium yurii subsp. margaretiae subsp. nov.: Test Tube.Brush Bacteria from Subgingival Dental Plaque
491	Cato	1985	Fusobacterium alocis sp. nov. and Fusobacterium sulci sp. nov. from the Human Gingival Sulcus
492	Siqueira	2003	Detection of Filifactor alocis in endodontic infections associated with different forms of periradicular diseases
493	Wakabayashi	1989	Flavobacterium branchiophila sp. nov. a Causative Agent of Bacterial Gill Disease of Freshwater Fishes
494	Bernardet	1986	Cutting a Gordian Knot: Emended Classification and Description of the Genus Flavobacterium, Emended Description of the Family Flavobacteriaceae, and Proposal of Flavobacterium hydatis norn. nov. (Basonym, Cytophaga aquatilis Strohl and Tait 1978)
495	Lewin	1969	A Classification of Flexibacteria
496	Hosoya	2007	Reclassification of Flexibacter aggregans (Lewin 1969) Leadbetter 1974 as a later heterotypic synonym of Flexithrix dorotheae Lewin 1970
497	Shinjo	1981	Proposal of Two Subspecies of Fusobacterium necrophorum (Flugge) Moore and Holdeman: Fusobacterium necrophorum subsp. necrophorum subsp. nov., nom. rev. (ex Flugge 1886), and Fusobacterium necrophorum subsp. funduliforme subsp. nov., nom. rev. (ex Hall6 1898)
498	Collins	1998	Gemella bergeriae sp. nov., Isolated from Human Clinical Specimens
499	Kilpper-Balz	1988	Transfer of Streptococcus morbillorum to the Genus Gemella as Gemella morbillorum comb. nov.
500	Collins	1998	Description of Gemella sanguinis sp. nov., Isolated from Human Clinical Specimens
501	Oren	1984	Halobacteroides halobius gen. nov., sp. nov., a Moderately Halophilic Anaerobic Bacterium from the Bottom Sediments ofthe Dead Sea
502	Peel	1997	Helcococcus kunzii as Sole Isolate from an Infected Sebaceous Cyst
503	Harper	2002	Helicobacter cetorum sp. nov., a Urease-Positive Helicobacter Species Isolated from Dolphins and Whales
504	Fox	2007	Isolation and Characterization of a Novel Helicobacter Species, “Helicobacter macacae,” from Rhesus Monkeys with and without Chronic Idiopathic Colitis”
505	Flores	2012	Hippea jasoniae sp. nov. and Hippea alviniae sp. nov., thermoacidophilic members of the class Deltaproteobacteria isolated from deep-sea hydrothermal vent deposits
506	Eggerth	1935	The Gram-positive Non-spore-bearing Anaerobic Bacilli of Human Feces
507	Iino	2010	Ignavibacterium album gen. nov., sp. nov., a moderately thermophilic anaerobic bacterium isolated from microbial mats at a terrestrial hot spring and proposal of Ignavibacteria classis nov., for a novel lineage at the periphery of green sulfur bacteria
508	Moore	1994	Oribaculum catoniae gen. nov., sp. nov.; Catonella morbi gen. nov., sp. nov.; Hallella seregens gen. nov., sp. nov.; Johnsonella ignava gen. nov., sp. nov.; and Dialister pneumosintes gen. nov., comb. nov., nom. rev., Anaerobic Gram-Negative Bacilli from the Human Gingival Crevice
509	Whitford	2001	Lachnobacterium bovis gen. nov., sp. nov., a novel bacterium isolated from the rumen and faeces of cattle
510	Morita	2010	Lactobacillus equicursoris sp. nov., isolated from the faeces of a thoroughbred racehorse
511	Endo	2010	Lactobacillus florum sp. nov., a fructophilic species isolated from flowers
512	Cousin	2012	Lactobacillus gigeriorum sp. nov., isolated from chicken crop
513	Cousin	2013	Lactobacillus pasteurii sp. nov. and Lactobacillus hominis sp. nov.
514	Chen	201	Lactobacillus pobuzihii sp. nov., isolated from pobuzihi (fermented cummingcordia)
515	Zou	2013	Lactobacillus shenzhenensis sp. nov., isolated from a fermented dairy beverage
516	Rodas	2006	Lactobacillus vini sp. nov., a wine lactic acid bacterium homofermentative for pentoses
517	Hellemond	1997	Leishmania infantum promastigotes have a poor capacity for anaerobic functioning and depend mainly on respiration for their energy generation
518	Vieira	1995	Amino acid uptake and intracellular accumulation in Leishmannia major promastigotes are largely determined by an H+-pump generated membrane potential
519	Ellenberger	1987	Biochemistry and Regulation of Folate and Methotrexate transport in Leishmania major
520	Naderer	2010	Evidence that Intracellular stages of Leishmania major utilize amino sugars as a major carbon source
521	Darling	1989	Carbon dioxide abolishes the reverse Pasteur effect in Leishmania major promastigotes
522	Chevrot	2008	Megamonas rupellensis sp. nov., an anaerobe isolated from the caecum of a duck
523	Podosokorskaya	2013	Characterization of Melioribacter roseus gen. nov., sp. nov., a novel facultatively anaerobic thermophilic cellulolytic bacterium from the class Ignavibacteria, and a proposal of a novel bacterial phylum Ignavibacteriae
524	Bellack	2011	Methanocaldococcus villosus sp. nov., a heavily flagellated archaeon that adheres to surfaces and forms cell–cell contacts
525	Takai	2004	Methanotorris formicicus sp. nov., a novel extremely thermophilic, methane-producing archaeon isolated from a black smoker chimney in the Central Indian Ridge
526	Burrgraf	1990	Methanococcus igneus sp. nov., a Novel Hyperthermophilic Methanogen from a Shallow Submarine Hydrothermal System
527	Leach	1973	Further Studies on Classification of Bovine Strains of Mycoplasmatales, with Proposals for New Species, Acholeplasma modicum and Mycoplasma alkalescens
528	Tully	1972	Synonymy of Mycoplasma arginini and Mycoplasma leonis
529	DaMassa	1994	Mycoplasma auris sp. nov., Mycoplasma cottewii sp. nov., and Mycoplasma yeatsii sp. nov., New Sterol-Requiring Mollicutes from the External Ear Canals of Goats
530	Jordan	1981	Isolation and Characterization of Mycoplasma columbium and Mycoplasma columborale, Two New Species from Pigeons
531	Rosendal	1973	Mycoplasma cynos, a New Canine Mycoplasma Species
532	Jordan	1982	Characterization and Taxonomic Description of Five Mycoplasma Serovars (Serotypes) of Avian Origin and Their Elevation to Species Rank and Further Evaluation of the Taxonomic Status of Mycoplasrna synoviae
533	Madden	1974	Mycoplasma moatsii, a New Species Isolated from Recently Imported Grivit Monkeys (Cercopithecus aethiops)
534	Tully	1974	Characterization of Some Caprine Mycoplasmas, with Proposals for New Species, Mycoplasma capricolum and M ycoplasma putvefaciens
535	Paek	2015	Myroides injenensis sp. nov., a new member isolated from human urine
536	Vancanneyt	1996	Reclassification of Flavobacterium odoraturn (Stutzer 1929) Strains to a New Genus, Myroides, as Myroides odoratus comb. nov. and Myroides odoratimimus sp. nov.
537	Kurtzman	2011	The Yeasts: A Taxonomic Study
538	Brew	1990	The rapid nitrosation of 2,3‐diaminonaphthalene by gastric isolates of Neisseria subflava
539	Nagai	2010	Alistipes indistinctus sp. nov. and Odoribacter laneus sp. nov., common members of the human intestinal microbiota isolated from faeces
540	Iino	2007	Oscillibacter valericigenes gen. nov., sp. nov., a valerate-producing anaerobic bacterium isolated from the alimentary canal of a Japanese corbicula clam
541	Takayuki	1983	Transfer of Peptococcus indolicus, Peptococcus asaccharolyticus, Peptococcus prevotii, and Peptococcus magnus to the Genus Peptostreptococcus and Proposal of Peptostreptococcus tetradius sp. nov.
542	Jung	2014	Peptoniphilus rhinitidis sp. nov., isolated from specimens of chronic rhinosinusitis
543	Downie	2006	Transport of nucleosides across the Plasmodium falciparum parasite plasma membrane has characteristics of PfENT1
544	Sherman	1979	Biochemistry of Plasmodium (Malarial Parasites)
545	Vander-Jagt	1990	D-Lactate production in erythrocytes infected with Plasmodium falciparum
546	Lwoff	1951	Biochemistry and Physiology of Protozoa
547	Lewis-Hughes	1984	In Vitro culture of plasmodium yoelii blood stages
548	Gosink	1998	Polaribacter gen. nov., with three new species, P. irgensii sp. nov., P. franzmannii sp. nov. and P. filamentus sp. nov., gas vacuolate polar marine bacteria of the C'ophaga- Flavobacterium-Bacteroides group and reclassificationof 'Flectobacillusglomeratus as Polaribacter glomeratus comb. nov.
549	Chen	2005	Proteiniphilum acetatigenes gen. nov., sp. nov., from a UASB reactor treating brewery wastewater
550	Dobson	1993	Direct Sequencing of the Polymerase Chain Reaction- Amplified 16s rRNA Gene of Flavobacten'um gondwanense sp. nov. and Flavobacten'um salegens sp. nov., Two New Species from a Hypersaline Antarctic Lake
551	Duncan	2006	Proposal of Roseburia faecis sp. nov., Roseburia hominis sp. nov. and Roseburia inulinivorans sp. nov., based on isolates from human faeces
552	Chandel	2011	Bioconversion of pentose sugars into ethanol: A review and future directions
553	Senac	1990	Intermediary Metabolite Concentrations in Xylulose- and Glucose- Fermenting Saccharomyces cerevisiae Cells
554	Kradolfer	1982	Tryptophan degradation in Saccharomyces cerevisiae: Characterization of two aromatic aminotransferases
555	Brayant	1956	The characteristics of strains of Selenomonas isolated from bovine rumen contents
556	Whitcomb	1997	Spiroplasma chrysopicola sp. nov., Spiroplasma gladiatoris sp. nov., Spiroplasma helicoides sp. nov., and Spiroplasma tabanidicola sp. nov., from Tabanid (Diptera: Tabanidae) Flies
557	Williamson	1996	Spiroplasma diminutum sp. nov., from Culex annulus Mosquitoes Collected in Taiwan
558	Clark	1985	Spiroplasrna melliferum, a New Species from the Honeybee (Apis mellifera)
559	Whitcomb	1996	Spiroplasma syrphidicola sp. nov., from a Syrphid Fly (Diptera: Syrphidae)
560	Abalain-Colloc	1988	Spiroplasma taiwanense sp. nov. from Culex tritaeniorhynchus Mosquitoes Collected in Taiwan
561	Chesneau	1993	Staphylococcus pasteuri sp. nov., Isolated from Human, Animal, and Food Specimens
562	Whiley	1991	Emended Descriptions and Recognition of Streptococcus constellatus, Streptococcus intermedius, and Streptococcus anginosus as Distinct Species
563	Poyart	2002	Taxonomic dissection of the Streptococcus bovis group by analysis of manganese- dependent superoxide dismutase gene (sodA) sequences: reclassification of ‘Streptococcus infantarius subsp. coli’ as Streptococcus lutetiensis sp. nov. and of Streptococcus bovis biotype II.2 as Streptococcus pasteurianus sp. nov.
564	Schlegel	2003	Reappraisal of the taxonomy of the Streptococcus bovis/Streptococcus equinus complex and related species: description of Streptococcus gallolyticus subsp. gallolyticus subsp. nov., S. gallolyticus subsp. macedonicus subsp. nov. and S. gallolyticus subsp. pasteurianus subsp. nov.
565	Zbinden	2012	Streptococcus tigurinus sp. nov., isolated from blood of patients with endocarditis, meningitis and spondylodiscitis
566	Janssen	1999	Succinispira mobilis gen. nov., sp. nov., a succinate-decarboxylatinganaerobic bacterium
567	Chamkha	2001	Isolation of a cinnamic acid-metabolizing Clostridium glycolicum strain from oil mill wastewaters and emendation of the species description
568	Chen	2013	Tetrapisispora taiwanensis sp. nov. and Tetrapisispora pingtungensis sp. nov., two ascosporogenous yeast species isolated from soil
569	Ueda-Nishimura	1999	A new yeast genus, Tetrapisisporagen. nov.: Tetrapisisporairiornotensis sp. nov., Tetrapisisporananseiensis sp. nov. and Tetrapisisporaarboricolasp. nov., frornthe Nansei Islands, and reclassification of Kluyveromyces phaffii (van der Walt) van der Walt as Tetrapisispora phaffii comb. nov.
570	Van der Walt	1963	Fabospora phaffii sp.n.
571	Lee	1993	Taxonomic Distinction of Saccharolytic Thermophilic Anaerobes: Description of Thermoanaerobacteriumxylanolyticumgen. nov.,sp. nov., and Thermoanaerobacterium saccharolyticum gen. nov., sp~. nov.;Reclassificationof Thermoanaerobiumbrockii,Clostridium thermosulfurogenes, and Clostridium thermohydrosulfiricum ElO0-69 as Thermoanaerobacter brockii comb. nov., Thermoanaerobacteriumthermosulfurigenescomb. nov.,and Thermoanaerobacter thermohydrosulfuricus comb. nov., Respectively; and Transfer of Clostridium thermohydrosulfuricum 39E to Thermoanaerobacter ethanolicus
572	Moussard	2004	Thermodesulfatator indicus gen. nov., sp. nov., a novel thermophilic chemolithoautotrophic sulfate-reducing bacterium isolated from the Central Indian Ridge
573	Hamilton-Brehm	2013	Thermodesulfobacterium geofontis sp. nov., a hyperthermophilic, sulfate-reducing bacterium isolated from Obsidian Pool, Yellowstone National Park
574	Mori	2003	A novel lineage of sulfate-reducing microorganisms: Themodesulfobiaceae farm. no., Thermodesulfobium narugense, gen. nov., sp. nov., a new thermophilic isolate from a hot spring
575	Chung	2000	Thermus igniterrae sp. nov. and Thermus antranikianii sp. nov., two new species from Iceland
576	Bjornsdottir	2009	Thermus islandicus sp. nov., a mixotrophic sulfur-oxidizing bacterium isolated from the Torfajokull geothermal area
577	Williams	1996	Thermus oshimai sp. nov., Isolated from Hot Springs in Portugal, Iceland, and the Azores, and Comment on the Concept of a Limited Geographical Distribution of Themus Species
578	Stanton	1980	Treponema bryantii sp. nov., a Rumen Spirochete that Interacts with Cellulolytic Bacteria
579	Graber	2004	Description of Treponema azotonutricium sp. nov. and Treponema primitia sp. nov., the First Spirochetes Isolated from Termite Guts
580	Cwyk	1979	Treponema succinifaciens sp. nov., an Anaerobic spirochete from the swine intestine
581	Heyworth	1984	Pyrimidine metabolism in Trichomonas vaginalis
582	Beach	1990	Fatty acid and sterol metabolism of cultured Trichomonas vaginalis and Tritichomonas foetus
583	Petrin	1998	Clinical and Microbiological Aspects of Trichomonas vaginalis
584	Mack	1980	End products of carbohydrate metabolism in Trichomonas vaginalis
585	Muller	2012	Biochemistry and Evolution of Anaerobic Energy Metabolism in Eukaryotes
586	Linstead	1983	The pathway of arginine catabolism in the parasitic flagellate Trichomonas vaginalis
587	Tsukahara	1961	Respiratory metabolism of trichomonad vaginalis
588	Heyworth	1982	Purine metabolism in Trichomonas vaginalis
589	Yarlett	1988	Polyamine biosynthesis and inhibition in Trichomonas vaginalis
590	Kleydman	2004	Production of ammonia by Tritrichomonas foetus and Trichomonas vaginalis
591	Chapman	1999	Hydrogen peroxide is a product of oxygen consumption by Trichomonas vaginalis
592	Ninomiya	1952	The metabolism of Trichomonas vaginalis, with comparative aspects of Trichomonads
593	Lehker	1999	Trichomonad invasion of the mucous layer requires adhesions, mucinases, and motility
594	Ginger	2007	Comparative genomics of trypanosome metabolism
595	Rogerson	1980	Catabolic metabolic in Trypanosoma cruzi
596	Bringaud	2006	Energy metabolism of typanosomatids: Adaptation to available carbon sources
597	Silber	2002	Active Transport of L-Proline in Trypanosoma cruzi
598	Taylor	2008	Validation of spermidine synthase as a drug target in African trypanosomes
599	Oliveira	2000	Inositol metabolism in Trypanosoma cruzi: Potential target for chemotherapy against chagas disease
600	Silber	2005	Amino Acid Metabolic Routes in Trypanosoma cruzi: Possible Therapeutic Targets Against Chagas’ Disease
601	Bosshard	2002	Turicibacter sanguinis gen. nov., sp. nov., a novel anaerobic, Gram-positive bacterium
602	Chung	2009	Varibaculum cambriense Infections in Hong Kong, China, 2006
603	Denariaz	1989	A Halophilic Denitrifier, Bacillus halodenitrificans sp. nov.
604	Yoon	2004	Transfer of Bacillus halodenitrificans Denariaz et al. 1989 to the genus Virgibacillus as Virgibacillus halodenitrificans comb. nov.
605	Sharpe	1972	Some Slime-Forming Heterofermentative Species of the Genus Lactobacillus
606	Choi	2002	Weissella kimchii sp. nov., a novel lactic acid bacterium from kimchi
607	Tohno	2014	Aerococcus vaginalis sp. nov., isolated from the vaginal mucosa of a beef cow, and emended descriptions of Aerococcus suis, Aerococcus viridans, Aerococcus urinaeequi, Aerococcus urinaehominis, Aerococcus urinae, Aerococcus christensenii and Aerococcus sanguinicola
608	Deibel	1960	Comparative study of Gaffkya homari, Aerococcus viridans, tetrad-forming cocci from meat curing brines, and the genus Pediococcus
609	Sakamoto	2009	Butyricimonas synergistica gen. nov., sp. nov. and Butyricimonas virosa sp. nov., butyric acid-producing bacteria in the family ‘Porphyromonadaceae’ isolated from rat faeces
610	Sakamoto	2014	Butyricimonas faecihominis sp. nov. and Butyricimonas paravirosa sp. nov., isolated from human faeces, and emended description of the genus Butyricimonas
611	Cooke	1927	A type of urea-splitting bacterium found in the human intestinal tract
612	Collins	1987	Transfer of Brevibacterium ammoniagenes (Cooke and Keith) to the Genus Corynebacterium as Corynebacterium ammoniagenes comb. nov.
613	Wilkins	1974	Eubacterium plexicaudatum sp. nov., an Anaerobic Bacterium with a sub polar tuft of flagella, isolated from a mouse cecum
614	Dent	1982	Lactobacillus animalis sp. nov., a New Species ofLactobacillus from the Alimentary Canal of Animals
615	Wood	1992	Genera of Lactic Acid Bacteria
616	Schleifer	1983	Elevation of Staphylococcus sciuri subsp. lentus (Kloos et al.) to Species Status: Staphylococcus lentus (Kloos et al.) comb. nov.
617	Cook	1994	Emendation of the Description of Acidaminococcus fermentans, a trans-Aconitate- and Citrate-Oxidizing Bacterium
618	Miller	1970	Nutritional Requirements for Growth of Aerococcus viridans
619	Bergaust	2008	Transcription and activities of NOx reductases in Agrobacterium tumefaciens: the influence of nitrate, nitrite and oxygen availability; Agrobacterium tumefaciens C58 Uses ActR and FnrN To Control nirK and nor Expression
620	Weelink	2008	Alicycliphilus denitrificans gen. nov., sp. nov., a cyclohexanol-degrading, nitrate-reducing b-proteobacterium; Isolation and Characterization of Alicycliphilus denitrificans Strain BC, Which Grows on Benzene with Chlorate as the Electron Acceptor
621	Schwiertz	2002	Anaerostipes caccae gen. nov., sp. nov., a New Saccharolytic, Acetate-utilising, Butyrate-producing Bacterium from Human Faeces
622	Karlsson	2011	Prospects for systems biology and modeling of the gut microbiome
623	Sirotek	2004	Fermentation of pectin and glucose, and activity of pectin-degrading enzymes in the rabbit caecal bacterium Bacteroides caccae
624	Christophersen	2013	Xylo-Oligosaccharides and Inulin Affect Genotoxicity and Bacterial Populations Differently in a Human Colonic Simulator Challenged with Soy Protein
625	Gibson	1993	Sulphate reducing bacteria and hydrogen metabolism in the human large intestine
626	Tannock	1977	Characteristics of Bacteroides Isolates from the Cecum of Conventional Mice
627	Reilly	1980	The carbon dioxide requirements of anaerobic bacteria
628	Balamurugan	2008	Real-time polymerase chain reaction quantification ofspecific butyrate-producing bacteria,Desulfovibrio andEnterococcus faecalis in the feces of patients withcolorectal cancer
629	Vandamme	1994	New Perspectives in the Classification of the Flavobacteria: Description of Chryseobacterium gen. nov., Bergeyella gen. nov., and Empedobacter norn. rev.
630	Holmes	1986	Weeksella zoohelcum sp. nov. (Formerly Group IIj), from Human Clinical Specimens
631	Goodfellow	2012	Bergey's Manual of Systematic Bacteriology, Volume 5: The Actinobacteria
632	Stanton	1991	Reclassification of Treponema hyodysenteriae and Treponema innocens in a New Genus, Serpula gen. nov., as Serpula hyodysenteriae comb. nov. and Serpula innocens comb. nov.
633	Donohoe	2011	The microbiome and butyrate regulate energy metabolism and autophagy in the mammalian colon
634	Fardeau	2004	Isolation from oil reservoirs of novel thermophilic anaerobes phylogenetically related to Thermoanaerobacter subterraneus: reassignment of T. subterraneus, Thermoanaerobacter yonseiensis, Thermoanaerobacter tengcongensis and Carboxydibrachium pacificum to Caldanaerobacter subterraneus gen. nov., sp. nov., comb. nov. as four novel subspecies
635	Colina	1996	Evidence for Degradation of Gastrointestinal Mucin by Candida albicans Secretory Aspartyl Proteinase
636	Leadbetter	1979	Capnocytophaga:New Genus of Gram-Negative Gliding Bacteria I. General Characteristics, Taxonomic Considerations and Significance
637	Pfennig	1968	Chlorobium phaeobacteroides nov. spec. und C. phaeovibrioides nov. spec.
638	Bergstein	1979	Uptake and metabolism of organic compounds by Chlorobium phaeobacteroides isolated from Lake Kinneret
639	Hofman	1985	Ecological significance of acetate assimilation by Chlorobium phaeobacteroides
640	Weber	2006	Anaerobic Nitrate-Dependent Iron(II) Bio-Oxidation by a Novel Lithoautotrophic Betaproteobacterium, Strain 2002
641	Srivastava	2003	Understanding Bacteria (Table 6.2 Type of fermentative pathway in different bacteria)
642	Fuchs	1957	The Nutritional Requirements of Clostridium perfringens
643	Steer	2001	Clostridium hathewayi sp. nov., from Human Faeces
644	Hall	1927	Bacillus sordellii, a cause of malignant edema in man
645	Ramachandran	2008	Hydrogen production and end-product synthesis patterns by Clostridium termitidis strain CT1112 in batch fermentation cultures with cellobiose or a-cellulose
646	Salminen	2004	Lactic Acid Bacteria: Microbiological and Functional Aspects, Third Edition
647	Mackie	1991	Lipid metabolism in anaerobic ecosystems
648	Widdel	1977	A New Anaerobic, Sporing, Acetate-Oxidizing, Sulfate-Reducing Bacterium, Desulfotomaculum (emend.)acetoxidans
649	Junier	2010	The genome of the Gram-positive metal- and sulfate-reducing bacterium Desulfotomaculum reducens strain MI-1
650	Gibson	1988	Occurrence of sulphate-reducing bacteria in human faeces and the relationship of dissimilatory sulphate reduction to methanogenesis in the large gut
651	Sakaguchi	2002	Desulfovibrio magneticus sp. nov., a novel sulfate-reducing bacterium that produces intracellular single-domain-sized magnetite particles
652	Zellner	1989	Desulfovibrio simplex spec. nov., a new sulfate-reducing bacterium from a sour whey digester
653	Wolfe	2005	The acetate switch
654	Mackie	1979	Changes in Lactate-Producing and Lactate-Utilizing Bacteria in Relation to pH in the Rumen of Sheep During Stepwise Adaptation to a High-Concentrate Diet
655	Duncan	2002	Growth requirements and fermentation products of Fusobacterium prausnitzii, and a proposal to reclassify it as Faecalibacterium prausnitzii gen. nov., comb. nov.
656	Scheifinger	1973	Propionate formation from cellulose and soluble sugars by combined cultures of Bacteroides succinogenes and Selenomonas ruminantium
657	Collins	1994	The phylogeny of the genus Clostridium: proposal of five new genera and eleven new species combinations.
658	Van Trappen	2004	Flavobacterium degerlachei sp. nov., Flavobacterium frigoris sp. nov. and Flavobacterium micromati sp. nov., novel psychrophilic bacteria isolated from microbial mats in Antarctic lakes
659	Zhilina	1992	Ecology, Physiology and Taxonomy Studies on a New Taxon of Haloanaerobiaceae, Haloincola saccharolytica gen. nov., sp. nov.
660	Collins	1993	Phylogenetic Analysis of Some Aerococcus-Like Organisms from Clinical Sources: Description of Helcococcus kunzii gen. nov., sp. nov.
661	Vandamme	1991	Revision of Campylobacter, Helicobacter, and Wolinella Taxonomy: Emendation of Generic Descriptions and Proposal of Arcobacter gen. nov.
662	De Maesschalck	2014	Faecalicoccus acidiformans gen. nov., sp. nov., isolated from the chicken caecum, and reclassification of Streptococcus pleomorphus (Barnes et al. 1977), Eubacterium biforme (Eggerth 1935) and Eubacterium cylindroides (Cato et al. 1974) as Faecalicoccus pleomorphus comb. nov., Holdemanella biformis gen. nov., comb. nov. and Faecalitalea cylindroides gen. nov., comb. nov., respectively, within the family Erysipelotrichaceae
663	Hedberg	2012	Lachnoanaerobaculum gen. nov., a new genus in the Lachnospiraceae: characterization of Lachnoanaerobaculum umeaense gen. nov., sp. nov., isolated from the human small intestine, and Lachnoanaerobaculum orale sp. nov., isolated from saliva, and reclassification of Eubacterium saburreum (Prevot 1966) Holdeman and Moore 1970 as Lachnoanaerobaculum saburreum comb. nov.
664	Gummalla	1999	Tryptophan Catabolism by Lactobacillus casei and Lactobacillus helveticus Cheese Flavor Adjuncts
665	Ishii	1999	Identification of Compounds Causing Symbiotic Growth of Lactobacillus paracasei subsp. tolerans and Kluyveromyces marxianus var. lactis in Chigo, Inner Mongolia, China
666	Teusink	2005	In silico reconstruction of the metabolic pathways of Lactobacillus plantarum: comparing predictions of nutrient requirements with those from growth experiments.
667	Golbach	2007	Adaptation of Lactobacillus rhamnosus riboflavin assay to microtiter plates
668	Rogosa	1953	Species differentiation of oral lactobacilli from man including description of Lactobacillus salivarius nov spec and lactobacillus Cellobiosus nov spec.
669	Embley	1989	Lactobacillus vaginalis sp. nov. from the Human Vagina
670	Flahaut	2013	Genome-scale metabolic model for Lactococcus lactis MG1363 and its application to the analysis of flavor formation
671	Liu	2011	Molecular Detection of Human Bacterial Pathogens
672	Haroun	1982	Reclassification of Bacteroides hypermegas (Harrison and Hansen) in a New Genus Megamonas, as Megamonas hypermegas comb. nov.
673	Bailey	1982	Reclassification of ‘Streptococcus pluton’ (White)in a new genus Melissococcus, as Melissococcus pluton nom. rev.; comb. nov.
674	Garrity	2001	Bergey's Manual of Systematic Bacteriology: Volume One : The Archaea and the Deeply Branching and Phototrophic Bacteria
675	Bordbar	2010	Insight into human alveolar macrophage and m. tuberculosis interactions via metabolic reconstructions
676	Pastink	2009	Genome-scale model of Streptococcus thermophilus LMG18311 for metabolic comparison of lactic acid bacteria.
677	Lee	2013	Oscillibacter ruminantium sp. nov., isolated from the rumen of Korean native cattle
678	Larocque	2014	A curated C. difficile strain 630 metabolic network: prediction of essential targets and inhibitors
679	Stanton	1983	Roseburia cecicola gen. nov., sp. nov., a Motile, Obligately Anaerobic Bacterium from a Mouse Cecum
680	Gradel	1972	Fermentation of isolated pectin and pectin from intact forages by pure cultures of rumen bacteria.
681	Yoon	2013	Shewanella spp. Use Acetate as an Electron Donor for Denitrification but Not Ferric Iron or Fumarate Reduction
682	Ahmed	1988	Nutritional requirements of shigellae for growth in a minimal medium.
683	Slobodkin	1999	Thermoanaerobater siderophilus sp. nov., a novel dissimilarity Fe(III)-reducing, anaerobic, thermophilic bacterium
684	Alain	2010	Thermodesulfatator atlanticus sp. nov., a thermophilic, chemolithoautotrophic, sulfate- reducing bacterium isolated from a Mid-Atlantic Ridge hydrothermal vent
685	Jeanthon	2002	Thermodesulfobacterium hydrogeniphilum sp. nov., a thermophilic, chemolithoautotrophic, sulfate-reducing bacterium isolated from a deep-sea hydrothermal vent at Guaymas Basin, and emendation of the genus Thermodesulfobacterium
686	Nobre	1996	Transfer of Thermus ruber (Loginova et al. 1984), Themus silvans (Tenreiro et al. 1999, and Themus chliarophilus (Tenreiro et al. 1995) to Meiothemzus gen. nov. as Meiothermus ruber comb. nov., Meiothermus silvanus comb. nov., and Meiothermus chliarophilus comb. nov., Respectively, and Emendation of the Genus Thermus
687	Kane	1978	Early Detection and Identification of Trichophyton verrucosum
688	Hall	2003	Characterization of some actinomyces-like isolates from human clinical sources: description of Varibaculum cambriensis gen nov, sp nov.
689	Rogosa	1964	THE GENUS VEILLONELLA II. Nutritional Studies
690	Boyer	2013	Bile Formation and Secretion
691	Alnouti	2008	Quantitative-profiling of bile acids and their conjugates in mouse liver, bile, plasma, and urine using LC-MS/MS.
692	Birchenough	2015	New developments in goblet cell mucus secretion and function
693	Nakano	1997	Characterization of anaerobic fermentative growth of Bacillus subtilis: identification of fermentation end products and genes required for growth.
694	Bezkorovainy	1989	Biochemistry and Physiology of Bifidobacteria
695	Duerden	1989	A comparison of Bacteroides ureolyticus isolates ftom different clinical sources
696	Sarantinopoulos	2001	Citrate metabolism by Enterococcus faecalis FAIR-E 229
697	Kim	2005	Dissimilatory Fe(III) reduction by an electrochemically active lactic acid bacterium phylogenetically related to Enterococcus gallina um isolated from submerged soil
698	Jacobson	1997	Cellulase activity of Leishmania major in the sandfly vector and in culture.
699	Scherer	1981	Effect of trace elements and vitamins on the growth of Methanosarcina barkeri
700	Boden	2012	Emended description of the genus Methylophaga Janvier et al. 1985
701	Bowman	1993	Revised Taxonomy of the Methanotrophs: Description of Methylobacter gen. nov., Emendation of Methylococcus, Validation of Methylosinus and Methylocystis Species, and a Proposal that the Family Methylococcaceae Includes Only the Group I Methanotrophs
702	Cato	1983	Synonymy of Strains of “Lactobacillus acidophilus” Group A2 (Johnson et 81. 1980) with the Type Strain of Lactobacillus crispatus (Brygoo and Aladame 1953) Moore and Holdeman 1970
703	Chervaux	2000	Physiological study of Lactobacillus delbrueckii subsp. bulgaricus strains in a novel chemically defined medium.
704	Helanto	2006	Characterization of genes involved in fructose utilization by Lactobacillus fermentum
705	Francl	2010	The PTS transporters of Lactobacillus gasseri ATCC 33323
706	Su	2011	Physiological and fermentation properties of Bacillus coagulans and a mutant lacking fermentative lactate dehydrogenase activity
707	De Clerck	2004	Polyphasic characterization of Bacillus coagulans strains, illustrating heterogeneity within this species, and emended description of the species.
708	Creczynski-Pasa	2004	Energetic metabolism of Chromobacterium violaceum
709	Takai	2006	Sulfurimonas paralvinellae sp. nov., a novel mesophilic, hydrogen- and sulfur-oxidizing chemolithoautotroph within the Epsilonproteobacteria isolated from a deep-sea hydrothermal vent polychaete nest, reclassification of Thiomicrospira denitrificans as Sulfurimonas denitrificans comb. nov. and emended description of the genus Sulfurimonas
710	Kopke	2013	Clostridium difficile is an autotrophic bacterial pathogen.
711	Christiansen	1996	Desulfitobacterium hafniense sp. nov., an Anaerobic, Reductively Dechlorinating Bacterium
712	Pfennig	1976	Desulfuromonas acetoxidans gen. nov. and sp. nov., a new anaerobic, sulfur-reducing, acetate-oxidizing bacterium.
713	Atherly	2014	Genotypic and Phenotypic comparison of Bacteroides ovatus, B. thetaiotaomicron, and B. xylanisolvens isolates obtained from cow, goat, human, and pig feces
714	Akagi	1967	Electron carries for the phosphoroclastic reaction of Desulfovibrio desulfuricans.
715	Lewis	2012	Host sialoglycans and bacterial sialidases: a mucosal perspective
716	Reddy	1983	Wheat straw hemicelluloses: Composition and fermentation by human colon bacteroides
717	Postgate	1966	Classification of Desulfovibrio species, the nonsporulating sulfate-reducing bacteria.
718	Warren	2005	Biochemical differentiation and comparison of Desulfovibrio species and other phenotypically similar genera.
719	Halpern	1961	Utilization of L-glutamic and 2-oxoglutaric acid as sole sources of carbon by Escherichia coli.
720	Hebert	2000	Nutritional requirements and nitrogen-dependent regulation of proteinase activity of Lactobacillus helveticus CRL 1062.
721	Horn	2005	Growth of Lactobacillus plantarum in media containing hydrolysates of fish viscera
722	Kalyuzhnaya	2006	Methylotenera mobilis gen. nov., sp. nov., an obligately methylamine-utilizing bacterium within the family Methylophilaceae
723	Helaszek	1991	Cellobiose uptake and metabolism by Ruminococcus flavefaciens.
724	Tong	2003	Streptococcus oligofermentans sp. nov., a novel oral isolate from caries-free humans
725	Feio	2004	Desulfovibrio alaskensis sp. nov., a sulphate- reducing bacterium from a soured oil reservoir
726	Niamsup	2003	Lactobacillus thermotolerans sp. nov., a novel thermotolerant species isolated from chicken faeces
727	Le Gall	1963	A New Species of Desulfovibrio
728	Collins	2000	An unusual Streptococcus from human urine, Streptococcus urinalis sp. nov.
729	Giebel	1990	Isolation of Mycoplasma moatsii from the Intestine of Wild Norway Rats (Rattus norvegicus)
730	Baele	2003	Lactobacillus ingluviei sp. nov., isolated from the intestinal tract of pigeons
731	Chander	2012	Phenotypic and molecular characterization of a novel strongly hemolytic Brachyspira species, provisionally designated “Brachyspira hampsonii ”
732	Schlegel	2000	Streptococcus infantarius sp. nov., Streptococcus infantarius subsp. infantarius subsp. nov. and Streptococcus infantarius subsp. coli subsp. nov., isolated from humans and food
733	Kloos	1976	Characterization of Staphylococcus sciuri sp.nov. and its subspecies
734	Robert	2007	Bacteroides cellulosilyticus sp. nov., a cellulolytic bacterium from the human gut microbial community
735	Chassard	2007	Characterization of the xylan-degrading microbial community from human faeces
736	Salyers	1979	Energy sources of major intestinal fermentative anaerobes
737	Attwood	1996	Clostridium proteoclasticum sp. nov., a Novel Proteolytic Bacterium from the Bovine Rumen
738	Gylswyk	1986	Description and Designation of a Neotype Strain of Eubacterium cellulosolvens (Cillobacterium cellulosolvens Bryant, Small, Bouma and Robinson) Holdeman and Moore
739	Lopez-Siles	2011	Cultured Representatives of Two Major Phylogroups of Human Colonic Faecalibacterium prausnitzii Can Utilize Pectin, Uronic Acids, and Host-Derived Substrates for Growth
740	Wallnofer	1967	Pathway of Propionate Formation in Bacteroides ruminicola
741	Zanoni	1987	Lactobacillus pentosus (Fred, Peterson, and Anderson) sp. nov., nom, rev.
742	Paterek	1988	Methanohalophilus mahii gen. nov. sp. nov. a Methylotrophic Halophilic Methanogen
743	Dedysh	2005	Methylocella Species Are Facultatively Methanotrophic
744	Cho	2003	Parvularcula bermudensis gen. nov., sp. nov., a marine bacterium that forms a deep branch in the a-Proteobacteria
745	Nicholson	2012	Host-Gut Microbiota Metabolic Interactions
746	Seetharam	1982	Absorption and Transport of Cobalamin (Vitamin B12)
747	Mekhjian	1979	Colonic Absorption of Unconjugated Bile Acids
748	Davila	2013	Re-print of “Intestinal luminal nitrogen metabolism: Role of the gut microbiota and consequences for the host”
749	Pal	2005	Hexachlorocyclohexane-degrading bacterial strains Sphingomonas paucimobilis B90A, UT26 and Sp+, having similar lin genes, represent three distinct species, Sphingobium indicum sp. nov., Sphingobium japonicum sp. nov. and Sphingobium francense sp. nov., and reclassification of [Sphingomonas] chungbukensis as Sphingobium chungbukense comb. nov.
750	Houghton	2015	Thermorudis pharmacophila sp. nov., a novel member of the class Thermomicrobia isolated from geothermal soil, and emended descriptions of Thermomicrobium roseum, Thermomicrobium carboxidum, Thermorudis peleae and Sphaerobacter thermophilus
751	Shiratori	2009	Clostridium clariflavum sp. nov. and Clostridium caenicola sp. nov., moderately thermophilic, cellulose-/cellobiose-digesting bacteria isolated from methanogenic sludge
752	Madden	1982	Isolation and Characterization of an Anaerobic, Cellulolytic Bacterium, Clostridium papyrosolvens sp. nov.
753	Warnick	2002	Clostridium phytofermentans sp. nov., a cellulolytic mesophile from forest soil
754	Motamedi	1998	Desulfovibrio aespoeensis sp. nov., a mesophilic sulfate-reducing bacterium from deep groundwater at Aspo hard rock laboratory, Sweden
755	Khelaifia	2011	Desulfovibrio piezophilus sp. nov., a piezophilic, sulfate-reducing bacterium isolated from wood falls in the Mediterranean Sea
756	Youn	2009	Characterization of the Dicarboxylate Transporter DctA in Corynebacterium glutamicum
757	Pokusaeva	2011	Carbohydrate metabolism in Bifidobacteria
758	Shah	1988	Proposal for Reclassification of Bacteroides asaccharolyticus, Bacteroides gingivalis, and Bacteroides endodontalis in a New Genus, Porphyromonas
759	Marshall	1967	Growth of Bacillus coagulans in Chemically Defined Media
760	Holländer	1975	Energy metabolism of some representatives of the Haemophilus group
761	Jensen	1986	Bacteroides pectinophilus sp. nov. and Bacteroides galacturonicus sp. nov.: Two Pectinolytic Bacteria from the Human Intestinal Tract
762	Kindberg	1987	Menaquinone production and utilization in germ-free rats after inoculation with specific organisms
763	Song	2004	Clostridium bartlettii sp. nov., isolatedfrom human faeces
764	Marquet	2009	Lactate has the potential to promote hydrogen sulphide formation in the human colon
765	Loubinoux	2002	Reclassification of the only species of the genus Desulfomonas, Desulfomonas pigra, as Desulfovibrio piger comb. nov.
766	Scott	2013	Prebiotic stimulation of human colonic butyrate-producing bacteria and bifidobacteria, in vitro
767	Shetty	2018	Reclassification of Eubacterium hallii as Anaerobutyricum hallii gen. nov., comb. nov., and description of Anaerobutyricum soehngenii sp. nov., a butyrate and propionate-producing bacterium from infant faeces
768	Cambell	1966	Desulfovibrio africanus sp. n., a New Dissimilatory Sulfate-reducing Bacterium
769	Abildgaard	2006	Desulfovibrio alkalitolerans sp. nov., a novel alkalitolerant, sulphate-reducing bacterium isolated from district heating water

**Online-only Table 3:** List of microbial species and host cell types in NJC19.

NCBI ID		Species name	Synonym
*	31971	Absiella dolichum	Eubacterium dolichum
	1511	Acetoanaerobium sticklandii	Clostridium sticklandii
	438	Acetobacter pasteurianus	
*	33952	Acetobacterium woodii	
	28187	Acetohalobium arabaticum	
	2148	Acholeplasma laidlawii	
	72556	Achromobacter piechaudii	
	85698	Achromobacter xylosoxidans	
	905	Acidaminococcus fermentans	
	53635	Acidimicrobium ferrooxidans	
	524	Acidiphilium cryptum	
	33059	Acidithiobacillus caldus	
	920	Acidithiobacillus ferrooxidans	
	28049	Acidothermus cellulolyticus	
	80867	Acidovorax avenae	
	47920	Acidovorax delafieldii	
	471	Acinetobacter calcoaceticus	
	40216	Acinetobacter radioresistens	
	67854	Actinobacillus succinogenes	
	103618	Actinomyces coleocanis	
	544580	Actinomyces oris	
	103621	Actinomyces urogenitalis	
	1656	Actinomyces viscosus	
	40567	Actinosynnema mirum	
*	1377	Aerococcus viridans	
	219314	Aeromicrobium marinum	
	644	Aeromonas hydrophila	
	645	Aeromonas salmonicida	
	56636	Aeropyrum pernix	
	714	Aggregatibacter actinomycetemcomitans	
	358	Agrobacterium tumefaciens	
	239935	Akkermansia muciniphila	
	179636	Alicycliphilus denitrificans	
*	214856	Alistipes finegoldii	
*	626932	Alistipes indistinctus	
*	1118061	Alistipes obesi	Bacteroidales bacterium ph8
*	328813	Alistipes onderdonkii	
	28117	Alistipes putredinis	
*	1288121	Alistipes senegalensis	
	328814	Alistipes shahii	
*	908612	Alistipes sp. HGB5	
*	671218	Alloprevotella rava	Prevotella sp. oral taxon 302
	76122	Alloprevotella tannerae	
	81468	Aminobacterium colombiense	
*	81412	Aminomonas paucivorans	
	39488	Anaerobutyricum hallii	Eubacterium hallii
	33029	Anaerococcus hydrogenalis	
	33032	Anaerococcus lactolyticus	
*	1287640	Anaerococcus obesiensis	
	33034	Anaerococcus prevotii	
	33036	Anaerococcus tetradius	
	33037	Anaerococcus vaginalis	
	105841	Anaerostipes caccae	
*	649756	Anaerostipes hadrus	Eubacterium hadrum
	169435	Anaerotruncus colihominis	
	2234	Archaeoglobus fulgidus	
	1382	Atopobium parvulum	
	1383	Atopobium rimae	
	82135	Atopobium vaginae	
	354	Azotobacter vinelandii	
	1390	Bacillus amyloliquefaciens	
	1392	Bacillus anthracis	
	1452	Bacillus atrophaeus	
	1413	Bacillus cellulosilyticus	
	1396	Bacillus cereus	
	79880	Bacillus clausii	
	1398	Bacillus coagulans	
	408580	Bacillus coahuilensis	
	86665	Bacillus halodurans	
	1402	Bacillus licheniformis	
	1404	Bacillus megaterium	
	1405	Bacillus mycoides	Bacillus weihenstephanensis
	79885	Bacillus pseudofirmus	
	64104	Bacillus pseudomycoides	
	1408	Bacillus pumilus	
	85683	Bacillus selenitireducens	
	1423	Bacillus subtilis	
	1428	Bacillus thuringiensis	
*	376804	Bacteroides barnesiae	
	47678	Bacteroides caccae	
	246787	Bacteroides cellulosilyticus	
*	626929	Bacteroides clarus	
	310298	Bacteroides coprocola	
	387090	Bacteroides coprophilus	
*	151276	Bacteroides coprosuis	
	357276	Bacteroides dorei	
	28111	Bacteroides eggerthii	
*	674529	Bacteroides faecis	
	338188	Bacteroides finegoldii	
*	626930	Bacteroides fluxus	
	817	Bacteroides fragilis	
*	376806	Bacteroides gallinarum	
	290053	Bacteroides helcogenes	
	329854	Bacteroides intestinalis	
*	204516	Bacteroides massiliensis	
*	291645	Bacteroides nordii	
*	626931	Bacteroides oleiciplenus	
	28116	Bacteroides ovatus	
	384638	Bacteroides pectinophilus	
	310297	Bacteroides plebeius	
*	392838	Bacteroides propionicifaciens	
*	310300	Bacteroides pyogenes	
	376805	Bacteroides salanitronis	
*	291644	Bacteroides salyersiae	
*	469589	Bacteroides sp. 2_1_33B	
	46506	Bacteroides stercoris	
	818	Bacteroides thetaiotaomicron	
	820	Bacteroides uniformis	
	821	Bacteroides vulgatus	
	371601	Bacteroides xylanisolvens	
*	1015	Bergeyella zoohelcum	
*	999	Bernardetia litoralis	Flexibacter litoralis
	1680	Bifidobacterium adolescentis	
	1683	Bifidobacterium angulatum	
	28025	Bifidobacterium animalis	
	1681	Bifidobacterium bifidum	
	1685	Bifidobacterium breve	
	1686	Bifidobacterium catenulatum	
	1689	Bifidobacterium dentium	
	78342	Bifidobacterium gallicum	
	216816	Bifidobacterium longum	
	28026	Bifidobacterium pseudocatenulatum	
*	1694	Bifidobacterium pseudolongum	
	35833	Bilophila wadsworthia	
	1322	Blautia hansenii	
	53443	Blautia hydrogenotrophica	
	40520	Blautia obeum	Ruminococcus obeum
*	33035	Blautia producta	Ruminococcus productus
*	1287055	Brachyspira hampsonii	
*	159	Brachyspira hyodysenteriae	
*	13264	Brachyspira innocens	
*	84377	Brachyspira intermedia	
*	84378	Brachyspira murdochii	
*	52584	Brachyspira pilosicoli	
	375	Bradyrhizobium japonicum	
	1393	Brevibacillus brevis	
	235	Brucella abortus	
	29459	Brucella melitensis	
	29461	Brucella suis	
	9	Buchnera aphidicola	
	152480	Burkholderia ambifaria	
	95486	Burkholderia cenocepacia	
	292	Burkholderia cepacia	
	337	Burkholderia glumae	
	87883	Burkholderia multivorans	
	60552	Burkholderia vietnamiensis	
*	544644	Butyricimonas synergistica	
	45851	Butyrivibrio crossotus	
	831	Butyrivibrio fibrisolvens	
	43305	Butyrivibrio proteoclasticus	
*	911092	Caldanaerobacter subterraneus	
	31899	Caldicellulosiruptor bescii	
	44001	Caldicellulosiruptor saccharolyticus	
*	693075	Caldisericum exile	
*	200415	Caldisphaera lagunensis	
	477976	Calditerrivibrio nitroreducens	
*	187145	Caldithrix abyssi	
*	515264	Caloramator australicus	
	291048	Caminibacter mediatlanticus	
*	195	Campylobacter coli	
*	199	Campylobacter concisus	
*	200	Campylobacter curvus	
*	196	Campylobacter fetus	
	824	Campylobacter gracilis	
*	76517	Campylobacter hominis	
	197	Campylobacter jejuni	
*	201	Campylobacter lari	
*	203	Campylobacter rectus	
*	204	Campylobacter showae	
*	28080	Campylobacter upsaliensis	
*	827	Campylobacter ureolyticus	
*	5476	Candida albicans	
*	42374	Candida dubliniensis	
*	5482	Candida tropicalis	
	186490	Candidatus Baumannia cicadellinicola	
*	28188	Capnocytophaga canimorsus	
*	28189	Capnocytophaga cynodegmi	
*	1017	Capnocytophaga gingivalis	
*	45242	Capnocytophaga granulosa	
*	1018	Capnocytophaga ochracea	
*	1019	Capnocytophaga sputigena	
*	300419	Catellicoccus marimammalium	
	100886	Catenibacterium mitsuokai	
	1711	Cellulomonas flavigena	
	29360	Cellulosilyticum lentocellum	Clostridium lentocellum
*	188913	Cetobacterium somerae	
	1096	Chlorobium phaeobacteroides	
	1108	Chloroflexus aurantiacus	
	536	Chromobacterium violaceum	
	545	Citrobacter koseri	
	67825	Citrobacter rodentium	
	133448	Citrobacter youngae	
	1496	Clostridioides difficile	Clostridium difficile, Peptoclostridium difficile
	1488	Clostridium acetobutylicum	
*	1137848	Clostridium arbusti	
	333367	Clostridium asparagiforme	
*	84023	Clostridium autoethanogenum	
	1520	Clostridium beijerinckii	
	208479	Clostridium bolteae	
	1491	Clostridium botulinum	
	1492	Clostridium butyricum	
	217159	Clostridium carboxidivorans	
*	36834	Clostridium celatum	
	1493	Clostridium cellulovorans	
*	358743	Clostridium citroniae	
*	1531	Clostridium clostridioforme	
*	179628	Clostridium colicanis	
	89152	Clostridium hiranonis	
	89153	Clostridium hylemonae	
*	1522	Clostridium innocuum	
	1534	Clostridium kluyveri	
	1535	Clostridium leptum	
	1538	Clostridium ljungdahlii	
	84026	Clostridium methylpentosum	
	1542	Clostridium novyi	Clostridium oedematiens
*	1501	Clostridium pasteurianum	
	1502	Clostridium perfringens	
*	169679	Clostridium saccharobutylicum	
	84030	Clostridium saccharolyticum	
*	36745	Clostridium saccharoperbutylacetonicum	
*	84031	Clostridium sartagoforme	Clostridium sartagoformum
	29347	Clostridium scindens	
*	97138	Clostridium sp. ASF356	
*	97139	Clostridium sp. ASF502	
	1509	Clostridium sporogenes	
	1512	Clostridium symbiosum	
	1513	Clostridium tetani	
*	219748	Clostridium tunisiense	
*	1519	Clostridium tyrobutyricum	
*	45497	Clostridium ultunense	
	74426	Collinsella aerofaciens	
	147207	Collinsella intestinalis	
	147206	Collinsella stercoris	
	285	Comamonas testosteroni	
	116085	Coprococcus catus	
	410072	Coprococcus comes	
	33043	Coprococcus eutactus	
*	1697	Corynebacterium ammoniagenes	
	1717	Corynebacterium diphtheriae	
	1718	Corynebacterium glutamicum	
	1747	Cutibacterium acnes	Propionibacterium acnes
	985	Cytophaga hutchinsonii	
	1299	Deinococcus radiodurans	
	118000	Denitrovibrio acetiphilus	
	453230	Desulfarculus baarsii	
	259354	Desulfatibacillum alkenivorans	
*	49338	Desulfitobacterium hafniense	
	2296	Desulfobacterium autotrophicum	
*	28223	Desulfobacula toluolica	
	894	Desulfobulbus propionicus	
*	873	Desulfocurvibacter africanus	Desulfovibrio africanus
	58138	Desulfofarcimen acetoxidans	
*	293256	Desulfohalovibrio alkalitolerans	Desulfovibrio alkalitolerans
	1565	Desulfotomaculum nigrificans	Desulfotomaculum carboxydivorans
	59610	Desulfotomaculum reducens	
*	58180	Desulfovibrio alaskensis	
	876	Desulfovibrio desulfuricans	
	878	Desulfovibrio fructosivorans	
*	879	Desulfovibrio gigas	
*	191026	Desulfovibrio hydrothermalis	
*	889	Desulfovibrio longus	
	184917	Desulfovibrio magneticus	
*	63560	Desulfovibrio oxyclinae	
	901	Desulfovibrio piger	
	880	Desulfovibrio salexigens	
*	42252	Desulfovibrio termitidis	
	881	Desulfovibrio vulgaris	
*	427923	Desulfurivibrio alkaliphilus	
*	64160	Desulfurobacterium thermolithotrophum	
	891	Desulfuromonas acetoxidans	
*	427926	Dethiobacter alkaliphilus	
	218538	Dialister invisus	
	39486	Dorea formicigenerans	Eubacterium formicigenerans
	88431	Dorea longicatena	
*	156974	Dysgonomonas gadei	
*	163665	Dysgonomonas mossii	
	84112	Eggerthella lenta	
*	5802	Eimeria tenella	
*	1117645	Elizabethkingia anophelis	
*	238	Elizabethkingia meningoseptica	
*	247	Empedobacter brevis	
*	312279	Emticicia oligotrophica	
	69218	Enterobacter cancerogenus	Enterobacter taylorae
	550	Enterobacter cloacae	
*	57732	Enterococcus asini	
*	33945	Enterococcus avium	
*	317735	Enterococcus caccae	
	37734	Enterococcus casseliflavus	
*	44008	Enterococcus cecorum	
*	1355	Enterococcus columbae	
*	44009	Enterococcus dispar	
*	53345	Enterococcus durans	
	1351	Enterococcus faecalis	
	1352	Enterococcus faecium	
	1353	Enterococcus gallinarum	
*	160453	Enterococcus gilvus	
*	155618	Enterococcus haemoperoxidus	
*	1354	Enterococcus hirae	
	246144	Enterococcus italicus	
*	71451	Enterococcus malodoratus	
*	155617	Enterococcus moraviensis	
*	53346	Enterococcus mundtii	
*	160454	Enterococcus pallens	
*	154621	Enterococcus phoeniculicola	
*	71452	Enterococcus raffinosus	
*	41997	Enterococcus saccharolyticus	
*	1356	Enterococcus sulfureus	
*	112904	Enterococcus villorum	Enterococcus porcinus
*	1547	Erysipelatoclostridium ramosum	Clostridium ramosum
	1648	Erysipelothrix rhusiopathiae	
	208962	Escherichia albertii	Escherichia/Shigella albertii
	562	Escherichia coli	
	564	Escherichia fergusonii	Escherichia/Shigella fergusonii
	253239	Ethanoligenens harbinense	
*	35517	Eubacterium brachy	
	29322	Eubacterium cellulosolvens	
	39485	Eubacterium eligens	
	1736	Eubacterium limosum	
*	97253	Eubacterium plexicaudatum	
*	39490	Eubacterium ramulus	
	39491	Eubacterium rectale	
	51123	Eubacterium saphenum	
	39492	Eubacterium siraeum	
	39496	Eubacterium ventriosum	
*	39498	Eubacterium yurii	
	853	Faecalibacterium prausnitzii	
	833	Fibrobacter succinogenes	
*	143361	Filifactor alocis	
	1260	Finegoldia magna	Peptostreptococcus magnus
*	271155	Flavobacterium antarcticum	
*	55197	Flavobacterium branchiophilum	
*	510946	Flavobacterium cauense	
*	996	Flavobacterium columnare	
*	1341165	Flavobacterium enshiense	
*	229204	Flavobacterium frigoris	
	986	Flavobacterium johnsoniae	
*	1401027	Flavobacterium limnosediminis	
*	96345	Flavobacterium psychrophilum	
*	498301	Flavobacterium rivuli	
*	329186	Flavobacterium saliperosum	
*	70993	Flexithrix dorotheae	
	849	Fusobacterium gonidiaformans	Fusobacterium gonidiiformans
	850	Fusobacterium mortiferum	Clostridium rectum
*	859	Fusobacterium necrophorum	
	851	Fusobacterium nucleatum	
	860	Fusobacterium periodonticum	
*	854	Fusobacterium russii	
	861	Fusobacterium ulcerans	
	856	Fusobacterium varium	
*	84136	Gemella bergeri	
*	29391	Gemella morbillorum	
*	84135	Gemella sanguinis	
	33940	Geobacillus thermodenitrificans	Bacillus thermodenitrificans
	225194	Geobacter bemidjiensis	
	313985	Geobacter lovleyi	
	28232	Geobacter metallireducens	
	35554	Geobacter sulfurreducens	
	351604	Geobacter uraniireducens	
	442	Gluconobacter oxydans	Gluconobacter uchimurae
	364410	Granulibacter bethesdensis	
	727	Haemophilus influenzae	
	729	Haemophilus parainfluenzae	
*	656519	Halanaerobium hydrogeniformans	
*	2331	Halanaerobium praevalens	
*	43595	Halanaerobium saccharolyticum	
	2238	Haloarcula marismortui	
	2242	Halobacterium salinarum	
*	42422	Halobacteroides halobius	
	2246	Haloferax volcanii	
*	40091	Helcococcus kunzii	
*	212	Helicobacter acinonychis	
*	37372	Helicobacter bilis	
*	56877	Helicobacter bizzozeronii	
*	123841	Helicobacter canadensis	
*	29419	Helicobacter canis	
*	138563	Helicobacter cetorum	
*	213	Helicobacter cinaedi	
*	214	Helicobacter felis	
*	215	Helicobacter fennelliae	
*	32025	Helicobacter hepaticus	
*	398626	Helicobacter macacae	
*	217	Helicobacter mustelae	
*	35818	Helicobacter pullorum	
	210	Helicobacter pylori	
*	104628	Helicobacter suis	
*	157268	Helicobacter winghamensis	
*	1279027	Hippea alviniae	
*	84405	Hippea maritima	
*	1735	Holdemanella biformis	Eubacterium biforme
	61171	Holdemania filiformis	
	35830	Hungateiclostridium cellulolyticum	Acetivibrio cellulolyticus
*	288965	Hungateiclostridium clariflavum	Clostridium clariflavum
	1515	Hungateiclostridium thermocellum	Clostridium thermocellum, Ruminiclostridium thermocellum
	154046	Hungatella hathewayi	Clostridium hathewayi
	53399	Hyphomicrobium denitrificans	
*	591197	Ignavibacterium album	
	160233	Ignicoccus hospitalis	
	167642	Ilyobacter polytropus	
	261299	Intestinibacter bartlettii	
*	43995	Johnsonella ignava	
	92945	Ketogulonicigenium vulgare	
	502	Kingella denitrificans	
	573	Klebsiella pneumoniae	
	244366	Klebsiella variicola	
	467210	Lachnoanaerobaculum saburreum	Eubacterium saburreum
*	140626	Lachnobacterium bovis	
	66219	Lachnoclostridium phytofermentans	Clostridium phytofermentans
*	89059	Lactobacillus acidipiscis	
	1579	Lactobacillus acidophilus	
	83683	Lactobacillus amylolyticus	
	1604	Lactobacillus amylovorus	Lactobacillus sobrius
*	1605	Lactobacillus animalis	
	227943	Lactobacillus antri	
	1580	Lactobacillus brevis	
	1581	Lactobacillus buchneri	
	1582	Lactobacillus casei	
	181675	Lactobacillus coleohominis	
*	1610	Lactobacillus coryniformis	
	47770	Lactobacillus crispatus	
*	28038	Lactobacillus curvatus	
	1584	Lactobacillus delbrueckii	
*	137357	Lactobacillus equi	
*	420645	Lactobacillus equicursoris	
*	1612	Lactobacillus farciminis	
	1613	Lactobacillus fermentum	
*	640331	Lactobacillus florum	
*	1614	Lactobacillus fructivorans	
	1596	Lactobacillus gasseri	
*	227942	Lactobacillus gastricus	
*	1203069	Lactobacillus gigeriorum	
	1587	Lactobacillus helveticus	
	1588	Lactobacillus hilgardii	
*	1203033	Lactobacillus hominis	
	147802	Lactobacillus iners	Lactobacillus sp. 7_1_47FAA
*	148604	Lactobacillus ingluviei	Lactobacillus thermotolerans
	109790	Lactobacillus jensenii	
	33959	Lactobacillus johnsonii	
*	267818	Lactobacillus kefiranofaciens	
*	176292	Lactobacillus malefermentans	
*	1618	Lactobacillus mali	
*	97478	Lactobacillus mucosae	
*	1622	Lactobacillus murinus	
	1632	Lactobacillus oris	
	1597	Lactobacillus paracasei	
*	872327	Lactobacillus pasteurii	
*	1589	Lactobacillus pentosus	
	1590	Lactobacillus plantarum	Lactobacillus arizonensis
*	449659	Lactobacillus pobuzihii	
	1598	Lactobacillus reuteri	
	47715	Lactobacillus rhamnosus	
*	231049	Lactobacillus rossiae	
	1623	Lactobacillus ruminis	
*	228229	Lactobacillus saerimneri	
	1599	Lactobacillus sakei	
	1624	Lactobacillus salivarius	
*	1625	Lactobacillus sanfranciscensis	
*	1231337	Lactobacillus shenzhenensis	
*	97137	Lactobacillus sp. ASF360	
*	152335	Lactobacillus suebicus	
	227945	Lactobacillus ultunensis	
	1633	Lactobacillus vaginalis	
*	194326	Lactobacillus versmoldensis	
*	238015	Lactobacillus vini	
	1358	Lactococcus lactis	
*	5671	Leishmania infantum	
*	5664	Leishmania major	
	40542	Leptotrichia buccalis	
	157692	Leptotrichia goodfellowii	
	157688	Leptotrichia hofstadii	
*	157691	Leptotrichia shahii	
*	157687	Leptotrichia wadei	
	33964	Leuconostoc citreum	
*	1244	Leuconostoc gelidum	
	136609	Leuconostoc kimchii	
	1245	Leuconostoc mesenteroides	
	1642	Listeria innocua	
	1639	Listeria monocytogenes	
	168384	Marvinbryantia formatexigens	
*	437897	Megamonas funiformis	
	158847	Megamonas hypermegale	
*	491921	Megamonas rupellensis	
	187326	Megasphaera micronuciformis	
*	1134405	Melioribacter roseus	
*	33970	Melissococcus plutonius	
	381	Mesorhizobium loti	
	43687	Metallosphaera sedula	
	83816	Methanobrevibacter ruminantium	
	2173	Methanobrevibacter smithii	
	83171	Methanocaldococcus fervens	
	67760	Methanocaldococcus infernus	
	2190	Methanocaldococcus jannaschii	
*	667126	Methanocaldococcus villosus	
	73913	Methanocaldococcus vulcanius	
	29291	Methanococcoides burtonii	
	42879	Methanococcus aeolicus	
	39152	Methanococcus maripaludis	
	2187	Methanococcus vannielii	
	2188	Methanococcus voltae	
	83984	Methanocorpusculum labreanum	
	2198	Methanoculleus marisnigri	
	2322	Methanohalobium evestigatum	
	2176	Methanohalophilus mahii	
	54120	Methanolacinia petrolearia	
	2320	Methanopyrus kandleri	
	2214	Methanosarcina acetivorans	
	2208	Methanosarcina barkeri	
	2209	Methanosarcina mazei	
	2317	Methanosphaera stadtmanae	
	475088	Methanosphaerula palustris	
	2203	Methanospirillum hungatei	
	145263	Methanothermobacter marburgensis	
	145262	Methanothermobacter thermautotrophicus	Methanobacterium thermautotrophicum
	155863	Methanothermococcus okinawensis	
	2180	Methanothermus fervidus	
	2224	Methanothrix thermoacetophila	
*	213185	Methanotorris formicicus	
*	2189	Methanotorris igneus	
	511746	Methylacidiphilum infernorum	
	105560	Methylibium petroleiphilum	
	405	Methylobacillus flagellatus	
	114616	Methylobacterium nodulans	
	199596	Methylocella silvestris	
	414	Methylococcus capsulatus	
	392484	Methylophaga thiooxydans	Methylophaga thiooxidans
	408	Methylorubrum extorquens	Methylobacterium dichloromethanicum, Methylobacterium chloromethanicum
	426	Methylosinus trichosporium	
	359408	Methylotenera mobilis	
	1270	Micrococcus luteus	
	52226	Mitsuokella multacida	
	1525	Moorella thermoacetica	
	480	Moraxella catarrhalis	
	1764	Mycobacterium avium	
	1769	Mycobacterium leprae	
	1773	Mycobacterium tuberculosis	
	1772	Mycolicibacterium smegmatis	
	2110	Mycoplasma agalactiae	
*	45363	Mycoplasma alkalescens	
	47687	Mycoplasma alligatoris	
*	2094	Mycoplasma arginini	
	2111	Mycoplasma arthritidis	
*	51363	Mycoplasma auris	
	28903	Mycoplasma bovis	
	2095	Mycoplasma capricolum	
*	114881	Mycoplasma columbinum	
	45361	Mycoplasma conjunctivae	
	50052	Mycoplasma crocodyli	
*	171284	Mycoplasma cynos	
	2115	Mycoplasma fermentans	
	2096	Mycoplasma gallisepticum	
	2097	Mycoplasma genitalium	
	29501	Mycoplasma haemofelis	
	2098	Mycoplasma hominis	
	2099	Mycoplasma hyopneumoniae	
	2100	Mycoplasma hyorhinis	
*	2116	Mycoplasma iowae	
	2105	Mycoplasma leachii	
*	171287	Mycoplasma moatsii	
	2118	Mycoplasma mobile	
	2102	Mycoplasma mycoides	
	28227	Mycoplasma penetrans	
	2104	Mycoplasma pneumoniae	
	2107	Mycoplasma pulmonis	
*	2123	Mycoplasma putrefaciens	
	2109	Mycoplasma synoviae	
*	51365	Mycoplasma yeatsii	
*	1183151	Myroides injenensis	
*	76832	Myroides odoratimimus	
*	256	Myroides odoratus	
*	27289	Naumovozyma dairenensis	
	484	Neisseria flavescens	
	485	Neisseria gonorrhoeae	
	488	Neisseria mucosa	
*	490	Neisseria sicca	
*	28449	Neisseria subflava	
*	626933	Odoribacter laneus	
	28118	Odoribacter splanchnicus	Bacteroides splanchnicus
*	351091	Oscillibacter valericigenes	
	847	Oxalobacter formigenes	
	1464	Paenibacillus larvae	
	1406	Paenibacillus polymyxa	
*	1505	Paeniclostridium sordellii	Clostridium sordellii
	823	Parabacteroides distasonis	Bacteroides distasonis
*	328812	Parabacteroides goldsteinii	Bacteroides goldsteinii
	387661	Parabacteroides johnsonii	
	46503	Parabacteroides merdae	Bacteroides merdae
	148447	Paraburkholderia phymatum	Burkholderia phymatum
*	1490	Paraclostridium bifermentans	Clostridium bifermentans
	266	Paracoccus denitrificans	
	208216	Parvularcula bermudensis	
	1254	Pediococcus acidilactici	
	19	Pelobacter carbinolicus	
	29543	Pelobacter propionicus	
	110500	Pelotomaculum thermopropionicum	
*	507750	Peptoniphilus duerdenii	
	54005	Peptoniphilus harei	Peptostreptococcus harei
*	33030	Peptoniphilus indolicus	
	33031	Peptoniphilus lacrimalis	Peptostreptococcus lacrimalis
*	1175452	Peptoniphilus rhinitidis	
*	1111134	Peptoniphilus sp. BV3C26	Clostridiales bacterium BV3C26
*	671216	Peptoniphilus sp. oral taxon 836	
*	1268254	Peptoniphilus timonensis	
	1261	Peptostreptococcus anaerobius	
	341694	Peptostreptococcus stomatis	
	82076	Picrophilus torridus	
*	5821	Plasmodium berghei	
*	5833	Plasmodium falciparum	
*	5850	Plasmodium knowlesi	
*	5855	Plasmodium vivax	
*	5861	Plasmodium yoelii	
*	37453	Polaribacter franzmannii	
*	531	Polaribacter irgensii	
	28123	Porphyromonas asaccharolytica	
	837	Porphyromonas gingivalis	
	419005	Prevotella amnii	
	242750	Prevotella bergensis	
	28125	Prevotella bivia	
	77095	Prevotella bryantii	
	28126	Prevotella buccae	
	28127	Prevotella buccalis	
	165179	Prevotella copri	
	28130	Prevotella disiens	
	189722	Prevotella marshii	
	28132	Prevotella melaninogenica	
	282402	Prevotella multiformis	
	28134	Prevotella oralis	
	28135	Prevotella oris	
	839	Prevotella ruminicola	
	228604	Prevotella salivae	
	386414	Prevotella timonensis	
	28137	Prevotella veroralis	
	1744	Propionibacterium freudenreichii	
*	294710	Proteiniphilum acetatigenes	
	584	Proteus mirabilis	
	102862	Proteus penneri	
	182210	Pseudodesulfovibrio aespoeensis	Desulfovibrio aespoeensis
*	879567	Pseudodesulfovibrio piezophilus	Desulfovibrio piezophilus
	106588	Pseudoflavonifractor capillosus	Bacteroides capillosus
	287	Pseudomonas aeruginosa	
	312306	Pseudomonas entomophila	
	294	Pseudomonas fluorescens	
	300	Pseudomonas mendocina	
	303	Pseudomonas putida	
	29438	Pseudomonas savastanoi	
	316	Pseudomonas stutzeri	
	317	Pseudomonas syringae	
*	251	Psychroflexus gondwanensis	
	638849	Pyramidobacter piscolens	
	13773	Pyrobaculum aerophilum	
	181486	Pyrobaculum calidifontis	
	29292	Pyrococcus abyssi	
	2261	Pyrococcus furiosus	
	53953	Pyrococcus horikoshii	
	329	Ralstonia pickettii	
	384	Rhizobium leguminosarum	
	1061	Rhodobacter capsulatus	
	1076	Rhodopseudomonas palustris	
	1085	Rhodospirillum rubrum	
	29549	Rhodothermus marinus	Rhodothermus obamensis
*	301301	Roseburia hominis	
	166486	Roseburia intestinalis	
	360807	Roseburia inulinivorans	
	2434	Roseobacter denitrificans	
*	29355	Ruminiclostridium cellobioparum	Clostridium termitidis, Ruminiclostridium cellobioparum subsp. termitidis
	1521	Ruminiclostridium cellulolyticum	Clostridium cellulolyticum
	29362	Ruminiclostridium papyrosolvens	Clostridium papyrosolvens
	1264	Ruminococcus albus	
	40518	Ruminococcus bromii	
	1265	Ruminococcus flavefaciens	
	33038	Ruminococcus gnavus	
	46228	Ruminococcus lactaris	
	33039	Ruminococcus torques	
	2287	Saccharolobus solfataricus	Sulfolobus solfataricus
*	4932	Saccharomyces cerevisiae	
	28901	Salmonella enterica	
	1660	Schaalia odontolytica	Actinomyces odontolyticus
	671224	Selenomonas artemidis	
	135080	Selenomonas flueggei	
	135083	Selenomonas noxia	
*	971	Selenomonas ruminantium	
	69823	Selenomonas sputigena	
	192073	Shewanella denitrificans	
	24	Shewanella putrefaciens	
	621	Shigella boydii	
	622	Shigella dysenteriae	
	623	Shigella flexneri	Escherichia/Shigella flexneri
	624	Shigella sonnei	
	382	Sinorhizobium meliloti	
	63612	Sodalis glossinidius	
	2057	Sphaerobacter thermophilus	
	332056	Sphingobium japonicum	
	154	Spirochaeta thermophila	
*	216933	Spiroplasma chrysopicola	
*	216936	Spiroplasma diminutum	
*	2134	Spiroplasma melliferum	
*	216945	Spiroplasma syrphidicola	
*	2145	Spiroplasma taiwanense	
	1280	Staphylococcus aureus	
	29388	Staphylococcus capitis	
	1281	Staphylococcus carnosus	
	1282	Staphylococcus epidermidis	
	1283	Staphylococcus haemolyticus	
	1290	Staphylococcus hominis	
*	42858	Staphylococcus lentus	
	28035	Staphylococcus lugdunensis	
*	45972	Staphylococcus pasteuri	
	283734	Staphylococcus pseudintermedius	
	29385	Staphylococcus saprophyticus	
	1292	Staphylococcus warneri	
	40324	Stenotrophomonas maltophilia	
	1311	Streptococcus agalactiae	
	1328	Streptococcus anginosus	
	113107	Streptococcus australis	
*	439220	Streptococcus caballi	
*	1329	Streptococcus canis	
*	76860	Streptococcus constellatus	
*	1333	Streptococcus criceti	
	45634	Streptococcus cristatus	Streptococcus oligofermentans
*	102886	Streptococcus didelphis	
	1317	Streptococcus downei	
	1334	Streptococcus dysgalactiae	
*	155680	Streptococcus entericus	
	1336	Streptococcus equi	
	1335	Streptococcus equinus	Streptococcus bovis
*	1345	Streptococcus ferus	
	315405	Streptococcus gallolyticus	
	1302	Streptococcus gordonii	
*	439219	Streptococcus henryi	
*	380397	Streptococcus ictaluri	
	102684	Streptococcus infantarius	
	68892	Streptococcus infantis	
*	1346	Streptococcus iniae	
*	1338	Streptococcus intermedius	
*	150055	Streptococcus lutetiensis	Streptococcus infantarius subsp. coli
*	1339	Streptococcus macacae	
*	59310	Streptococcus macedonicus	Streptococcus gallolyticus subsp. macedonicus
*	269666	Streptococcus marimammalium	
*	313439	Streptococcus massiliensis	
*	400065	Streptococcus merionis	
*	229549	Streptococcus minor	
	28037	Streptococcus mitis	
	1309	Streptococcus mutans	
	1303	Streptococcus oralis	Streptococcus tigurinus, Streptococcus oralis subsp. tigurinus
*	114652	Streptococcus orisratti	
*	82806	Streptococcus ovis	
	1318	Streptococcus parasanguinis	Streptococcus gallolyticus subsp. pasteurianus
*	1348	Streptococcus parauberis	
*	197614	Streptococcus pasteurianus	
	68891	Streptococcus peroris	
	1313	Streptococcus pneumoniae	
*	1340	Streptococcus porcinus	
*	257758	Streptococcus pseudopneumoniae	
	361101	Streptococcus pseudoporcinus	
	1314	Streptococcus pyogenes	
*	1341	Streptococcus ratti	
	1304	Streptococcus salivarius	
	1305	Streptococcus sanguinis	
*	1310	Streptococcus sobrinus	
*	1169673	Streptococcus sp. GMD4S	
	1307	Streptococcus suis	
	1308	Streptococcus thermophilus	
*	55085	Streptococcus thoraltensis	
	1349	Streptococcus uberis	
*	149016	Streptococcus urinalis	
	1343	Streptococcus vestibularis	
	1911	Streptomyces griseus	
	1916	Streptomyces lividans	
	1938	Streptomyces viridochromogenes	
	214851	Subdoligranulum variabile	
*	78120	Succinispira mobilis	
	2285	Sulfolobus acidocaldarius	
	39766	Sulfurimonas denitrificans	
	40545	Sutterella wadsworthensis	
	119484	Syntrophobacter fumaroxidans	
	51197	Syntrophobotulus glycolicus	
	863	Syntrophomonas wolfei	
	86170	Syntrophothermus lipocalidus	
	316277	Syntrophus aciditrophicus	
*	36841	Terrisporobacter glycolicus	Clostridium glycolicum
*	1071379	Tetrapisispora blattae	
*	113608	Tetrapisispora phaffii	
*	29323	Thermoanaerobacter brockii	
*	1757	Thermoanaerobacter ethanolicus	
*	1125974	Thermoanaerobacter indiensis	
*	108150	Thermoanaerobacter italicus	
*	583357	Thermoanaerobacter mathranii	
*	496866	Thermoanaerobacter pseudethanolicus	
*	106578	Thermoanaerobacter siderophilus	
*	1516	Thermoanaerobacter thermohydrosulfuricus	Clostridium thermohydrosulfuricum
*	46354	Thermoanaerobacter wiegelii	
*	28896	Thermoanaerobacterium saccharolyticum	
*	1517	Thermoanaerobacterium thermosaccharolyticum	
*	29329	Thermoanaerobacterium xylanolyticum	Thermoanaerobacter xylanolyticum
	2021	Thermobifida fusca	
*	1510	Thermoclostridium stercorarium	Clostridium stercorarium
	55802	Thermococcus barophilus	
	187878	Thermococcus gammatolerans	
	311400	Thermococcus kodakarensis	
	342948	Thermococcus onnurineus	
	172049	Thermococcus sibiricus	
*	501497	Thermodesulfatator atlanticus	
*	171695	Thermodesulfatator indicus	
*	1295609	Thermodesulfobacterium geofontis	
*	886	Thermodesulfobacterium thermophilum	
*	184064	Thermodesulfobium narugense	
	2303	Thermoplasma acidophilum	
	2336	Thermotoga maritima	
	2337	Thermotoga neapolitana	
*	271	Thermus aquaticus	
*	88189	Thermus igniterrae	
*	540988	Thermus islandicus	
*	56957	Thermus oshimai	
*	37636	Thermus scotoductus	
	274	Thermus thermophilus	
	36861	Thiobacillus denitrificans	
*	163	Treponema bryantii	
*	88058	Treponema primitia	
*	167	Treponema succinifaciens	
*	5722	Trichomonas vaginalis	
*	63417	Trichophyton verrucosum	
*	5691	Trypanosoma brucei	
*	5693	Trypanosoma cruzi	
*	154288	Turicibacter sanguinis	
	29361	Tyzzerella nexilis	Clostridium nexile
	134821	Ureaplasma parvum	
	2130	Ureaplasma urealyticum	
*	184870	Varibaculum cambriense	
	39777	Veillonella atypica	
	39778	Veillonella dispar	
	29466	Veillonella parvula	
	666	Vibrio cholerae	
	672	Vibrio vulnificus	
*	1482	Virgibacillus halodenitrificans	
*	1583	Weissella confusa	
	51229	Wigglesworthia glossinidia	
	844	Wolinella succinogenes	
	339	Xanthomonas campestris	
	2371	Xylella fastidiosa	
	542	Zymomonas mobilis	
		human colonocyte	
		human goblet cell	
		human hepatocyte	
*		mouse goblet cell	
*		mouse hepatocyte	
*		mouse intestinal cell	

*Denotes a species/host cell type that was not included in the predecessor, NJS16 (Sung *et al*., Nat. Commun. **8**, 15393 (2017)).

**Online-only Table 4:** List of small-molecule metabolites and macromolecules in NJC19.

Compound name		KEGG compound identifier
	D-Glucose (Glucose)	C00031
	Sucrose	C00089
	D-Fructose (Fructose)	C00095
	Glycerol	C00116
	D-Ribose (Ribose)	C00121
	D-Galactose	C00124
	N-Acetyl-D-Glucosamine (N-Acetylglucosamine)	C00140
	D-Mannose (Mannose)	C00159
	D-Xylose (Xylose)	C00181
	Cellobiose	C00185
	D-Glucuronic acid (D-Glucuronate)	C00191
	Maltose	C00208
	D-Arabinose (L-Arabinose, Arabinose, L-Arabinopyranose, L-Arabinofuranose)	C00216, C00259
	Lactose	C00243
	L-Sorbose (Sorbose)	C00247
	Isomaltose	C00252
	D-Gluconate (D-Gluconic acid, Gluconate)	C00257
	D-Glycerate (Glycerate, [R]-Glycerate, Glyceric acid)	C00258
	N-Acetylneuraminic acid (N-acetylneuraminate, Neu5Ac, Sialic acid)	C00270
	D-Xylulose (Xylulose, L-Xylulose)	C00310, C00312
	D-Glucosamine (Glucosamine)	C00329
	D-Galacturonate	C00333
	Xylitol	C00379
	D-Mannitol (Mannitol)	C00392
	Ribitol (Adonitol)	C00474
	D-Lyxose (L-Lyxose)	C00476, C01508
	Raffinose	C00492
	Erythritol	C00503
	L-Rhamnose (Rhamnose, D-Rhamnose)	C00507, C01684
	L-Arabitol (L-Arabinitol, D-Arabitol)	C00532, C01904
	D-Tagaturonate	C00558
	N-Acetyl-D-mannosamine	C00645
	Dextrin (Maltotriose, Maltodextrin, Maltohexaose, Maltotetraose, Maltooligosaccharide)	C00721, C01835, C01935, C01936, C02052
	L-Idonate	C00770
	D-Sorbitol (D-Glucitol, Sorbitol, Glucitol, L-Sorbitol, Sorbitol)	C00794, C01722
	D-Tagatose	C00795
	D-Fructuronate	C00905
	L-Fucose	C01019
	N-Acetylgalactosamine (N-Acetyl-D-galactosamine)	C01074
	Trehalose	C01083
	Stachyose	C01613
	Galactitol (Dulcitol)	C01697
	Palatinose (Isomaltulose)	C01742
	Cellotetraose (Cellohexaose, Cellopentaose, Cellotriose)	C02013, C06217, C06218, C06219
	D-Galactosamine (Galactosamine)	C02262
	Melibiose	C05402
	D-Psicose	C06468
	L-iduronate	C06472
	D-Turanose	C19636
	FOS (Fructooligosaccharide)	-
	XOS (Xylooligosaccharide)	-
	L-Glutamate (L-Glutamic acid, Glutamate, D-Glutamate)	C00025, C00217
	L-Glycine (Glycine)	C00037
	L-Alanine (D-Alanine, Alanine)	C00041, C00133
	L-Lysine (Lysine, D-Lysine)	C00047, C00739
	L-Aspartate (Aspartate, D-Aspartate)	C00049, C00402
	L-Arginine (Arginine)	C00062
	L-Glutamine (D-Glutamine, Glutamine)	C00064, C00819
	L-Serine (Serine, D-Serine)	C00065, C00740
	L-Methionine (D-Methionine)	C00073, C00855
	L-Ornithine (Ornithine)	C00077
	L-Tryptophan (Tryptophan)	C00078
	L-Phenylalanine (Phenylalanine, D-Phenylalanine)	C00079, C02265
	L-Tyrosine (Tyrosine)	C00082
	L-Cysteine (Cysteine, D-Cysteine)	C00097, C00793
	beta-alanine (3-Aminopropionate)	C00099
	L-Leucine (Leucine)	C00123
	L-Histidine (Histidine)	C00135
	L-Proline (Proline, D-Proline)	C00148, C00763
	L-Asparagine (Asparagine)	C00152
	L-Valine (Valine, D-Valine)	C00183, C06417
	L-Threonine (Threonine)	C00188
	L-Homoserine	C00263
	L-Carnitine (D-Carnitine)	C00318, C15025
	L-Isoleucine (Isoleucine)	C00407
	L-Selenocysteine	C05688
	Uracil	C00106
	Adenine	C00147
	Thymine	C00178
	Adenosine	C00212
	Thymidine	C00214
	Guanine	C00242
	Inosine	C00294
	Uridine	C00299
	Cytosine	C00380
	Xanthine	C00385
	Guanosine	C00387
	Cytidine	C00475
	Ascorbic acid (Vitamin C, L-Ascorbate, Ascorbate)	C00072
	Choline	C00114
	Biotin (Vitamin B7)	C00120
	Adenosylcobalamin (Vitamin B12, Cobamide coenzyme, Cob[II]alamin, Vitamin B12r, Cob[I]alamin, Vitamin B12s, Aquacobalamin, Cobalamin [III])	C00194
	Pyridoxal (Vitamin B6, Pyridoxine, Pyridoxamine, Vitamin B6)	C00250, C00314, C00534
	Niacin (Vitamin B3, Nicotinic acid, Nicotinate, Nicotinamide, Vitamin B3)	C00153, C00253
	Riboflavin (Vitamin B2)	C00255
	Thiamine (Vitamin B1, Thiamin)	C00378
	Retinol (Vitamin A)	C00473
	Folic acid (Folate, Vitamin B9)	C00504
	Retinoate	C00777
	Menaquinone (Vitamin K2)	C00828
	Pantothenic acid (Vitamin B5, Pantothenate)	C00864
	Phylloquinone (Vitamin K1)	C02059
	alpha-Tocopherol (Vitamin E, alpha-Tocotrienol)	C02477, C14155, C14153
	Arachidonate	C00219
	Cholecalciferol (Vitamin D3, Vitamin D2)	C05443, C05441
	Palmitate (Palmitic acid, Hexadecanoic acid, Hexadecanoate)	C00249
	1,2-diacylglycerol (1-acylglycerol, Monoacylglycerol, Monoglyceride, Monoacylglycerol, Diacylglycerol)	C00641, C01885, C00165
	Oleic acid ([9Z]-Octadecenoic acid)	C00712
	Lipoic acid (Lipoate)	C00725
	Stearic acid (Stearate, Octadecanoate)	C01530
	Decanoic acid (Decanoate)	C01571
	Caproate (Hexanoate)	C01585
	Linoleic acid (Omega-6 fatty acid)	C01595
	Pelargonic acid (Pelargonate, Nonanoic acid)	C01601
	Octanoate (Caprylic acid)	C06423
	Myristic acid (Tetradecanoic acid, Tetradecanoate)	C06424
	Arachidate (Arachidic acid)	C06425
	alpha-Linolenic acid (Omega-3 fatty acid, eicosapentaenoic acid)	C06427, C12083
	Acetate	C00033
	Propanoate (Propionate)	C00163
	Butyrate	C00246
	Valerate (Pentanoic acid, Pentanoate)	C00803
	Isobutyrate (2-Methylpropanoic acid)	C02632
	Isovalerate (3-Methylbutanoic acid)	C08262
	Methanol	C00132
	Inositol (myo-Inositol, Inositol 1-phosphate, Myo-inositol phosphate)	C00137, C01177
	Phenol	C00146
	Acetoin (3-hydroxybutanone, acetyl methyl carbinol, [R]-Acetoin)	C00466, C00810
	Ethanol	C00469
	Benzyl alcohol	C00556
	1,2-propanediol (Propene diol, Propylene glycol, [R]-1,2-propanediol, [R]-propane-1,2-diol, [S]-1,2-propanediol, [S]-propane-1,2-diol)	C00583, C02912, C02917
	1,2-Ethanediol (Ethylene glycol)	C01380
	Isopropanol (2-Propanol)	C01845
	1,3-Propanediol	C02457
	[R,R]-2,3-Butanediol ([R,R]-Butanediol, [S,S]-2,3-Butanediol, [S,S]-Butanediol)	C03044, C03046
	Propanol (n-propanol, 1-Propanol)	C05979
	Isobutyl alcohol (isobutanol, 2-Methyl-1-propanol)	C14710
	Butanol	C06142
	Pentanol	C16834
	Formaldehyde	C00067
	Acetaldehyde	C00084
	Benzaldehyde	C00261
	Propanal	C00479
	Putrescine	C00134
	Ethanolamine (Phosphatidylethanolamine)	C00189, C00350
	Methylamine (Monomethylamine)	C00218
	Spermidine	C00315
	Histamine	C00388
	Tryptamine	C00398
	Tyramine	C00483
	Dimethylamine	C00543
	Trimethylamine	C00565
	Spermine	C00750
	Trimethylamine N-oxide (Trimethylamine-N-oxide)	C01104
	Cadaverine	C01672
	Phenylethylamine	C02455
	Butylamine	C18706
	Urea	C00086
	Acetone	C00207
	Indole	C00463
	Toluene	C01455
	p-Cresol (4-methylphenol, 4-cresol)	C01468
	Anthranilate (o-amino-benzoic acid)	C00108
	Benzoate	C00180
	4-Aminobenzoate (para-aminobenzoic acid, PABA, p-Aminobenzoate)	C00568
	Indole-3-acetate (Indoleacetate)	C00954
	Ferulate	C01494
	Hippurate	C01586
	3-phenylpropionic acid (Phenylpropanoate, 3-phenylpropionate)	C05629
	Vanillate	C06672
	Phenylacetate	C07086
	Pyruvate	C00022
	alpha-ketoglutarate (2-oxoglutarate)	C00026
	Oxaloacetate	C00036
	Succinate	C00042
	Glyoxylate (Glyoxylic acid, Glyoxalate)	C00048
	Formate	C00058
	2-Oxobutyrate (Alpha-ketobutyrate, 2-Oxobutanoate)	C00109
	L-Malate ([S]-Malate, L-Malic acid, Malate, D-Malate, [R]-Malate)	C00149, C00497
	Fumarate	C00122
	Citrate	C00158
	Glycolate	C00160
	L-Lactate ([S]-Lactate, Lactate, D-Lactate, [R]-Lactate)	C00186, C00256
	Oxalate	C00209
	Taurine	C00245
	Orotic acid (Orotate)	C00295
	4-Aminobutyrate (GABA)	C00334
	Malonate	C00383
	cis-Aconitate (trans-Aconitate, trans-Aconitic acid)	C00417, C02341
	5-Aminovalerate	C00431
	Glutarate	C00489
	Itaconate	C00490
	Shikimate	C00493
	meso-Tartrate (meso-Tartaric acid, Tartrate, L-tartrate, L-Tartaric acid)	C00552, C00898
	Cholic acid (Cholate)	C00695
	Betaine (Glycine betaine)	C00719
	Crotonate (2-Butenoate, 2-Butenoic acid, Crotonic acid, 3-Methylacrylic acid)	C01771
	Glycocholate	C01921
	2-Aminobutyric acid (2-Aminobutyrate)	C02261, C02356
	Chenodeoxycholic acid (Chenodeoxycholate)	C02528
	Taurolithocholate	C02592
	Pimelate	C02656
	Lithocholic acid	C03990
	Deoxycholic acid	C04483
	Taurocholate	C05122
	Isethionate	C05123
	Taurodeoxycholate	C05463
	Glycodeoxycholate	C05464
	Taurochenodeoxycholate	C05465
	Glycochenodeoxycholate	C05466
	Adipate	C06104
	Glycolithocholate	C15557
	2-methylbutyrate (2-methylbutanoic acid)	C18319
	H2O2	C00027
	Zn2+ (Zinc)	C00038
	Sulfate (Sulfuric acid, Sulfite)	C00059, C00094
	Cu2+ (Copper)	C00070
	Ca2+ (Calcium)	C00076
	K+ (Potassium)	C00238
	Nitrate (NO3-)	C00244
	Bicarbonate (HCO3-, H2CO3, Carbonic acid, Carbonate)	C00288
	Mg2+ (Magnesium)	C00305
	Thiosulfate	C00320
	Dimethyl sulfide (Methyl sulfide)	C00580
	Cl− (Chloride)	C00698
	I− (Iodide)	C00708
	Na+ (Sodium)	C01330
	Selenite	C05684
	Fe3+ (Ferric ion, Fe2+, Ferrous ion)	C14819, C14818
	Sulfur (Elemental sulfur)	C00087
	CO2	C00011
	NH3 (Ammonia, NH4+, Ammonium)	C00014
	Carbon monoxide (CO)	C00237
	H2 (Hydrogen)	C00282
	H2S (HS-)	C00283
	Nitric Oxide (NO)	C00533
	Nitrite (NO2-)	C00088
	N2	C00697
	Methane (CH4)	C01438
	Dichloromethane (Methylene chloride)	C02271
	DNA RNA Polynucleotide	C00039, C00046, C00419
	Starch (Amylopectin, Amylose, 1,4-alpha-D-Glucan, Pullulan, Resistant starch, Glycogen)	C00317, C00369, C00480, C00718, C00182
	Polypeptide	C00403
	Triglyceride	C00422
	Chitin	C00461
	Mannan	C00464
	Hyaluronan (Hyaluronate, Hyaluronic acid)	C00518
	Arabinogalactan	C00569
	Chondroitin 4-sulfate (Chondroitin 6-sulfate)	C00634, C00635
	Pectin	C00714
	Cellulose (Beta-D-glucan)	C00760
	Fructan (Inulin, Levan)	C01355, C03323, C06215
	Galactan	C05796
	Mucin (Mucus Glycoprotein)	C02705
*	Hemicellulose, not specified	—
	Xylan	C00707
	Arabinan	C02474
	Hyodeoxycholic acid	—
	alpha-Muricholic acid	C17647
	beta-Muricholic acid	C17726
	omega-Muricholic acid	C17727
	Tauro-b-muricholic acid	—
	Tauroursodeoxycholic acid	C16868
	Ursodeoxycholic acid	C07880
	Indole-3-lactic acid	—
	Indole-3-carboxaldehyde	C08493
	7-Oxodeoxycholic acid	—
	7-Oxolithocholic acid	—
	Carnosine	C00386
	Glycylsarcosine	—
	Prolylglycine	—
	Glycyl-Phenylalanine	—
	Levomefolic acid	—
	H+	C00080
	Leucyl-leucine	C11332
	Alanylalanine	—
	Glycylproline	—
	Glycyl-leucine	C02155
	Leucylglycine	—
	L-Dopa	C00355
	Lysophosphatidylcholine (1-Lysophosphatidylcholine, 2-Lysophosphatidylcholine)	C04230, C04233
	Cholesterol	C00187
	Glycylglycine	C02037

*Denotes hemicellulose that is not yet specified as xylan, mannan, or others in NJC19.

**Online-only Table 5:** Degradation products of macromolecules in NJC19.

Macromolecule	Degradation product(s)	Remark
Arabinogalactan	D-Galactose, D-Arabinose (L-Arabinose, Arabinose, L-Arabinopyranose, L-Arabinofuranose)	
Cellulose (Beta-D-glucan)	D-Glucose (Glucose), Cellobiose, Cellotetraose (Cellohexaose, Cellopentaose, Cellotriose)	
Chitin	N-Acetyl-D-Glucosamine (N-Acetylglucosamine)	
Chondroitin 4-sulfate (Chondroitin 6-sulfate)	D-Glucuronic acid (D-Glucuronate), N-Acetylgalactosamine (N-Acetyl-D-galactosamine), Sulfate (Sulfuric acid, Sulfite)	
Fructan (Inulin, Levan)	D-Fructose (Fructose), FOS (Fructooligosaccharide)	
Galactan	D-Galactose	
Hemicellulose, not specified	D-Glucose (Glucose), D-Ribose (Ribose), D-Galactose, D-Mannose (Mannose), D-Xylose (Xylose), D-Glucuronic acid (D-Glucuronate), D-Arabinose (L-Arabinose, Arabinose, L-Arabinopyranose, L-Arabinofuranose), D-Galacturonate, L-Rhamnose (Rhamnose, D-Rhamnose), XOS (Xylooligosaccharide)	
Hyaluronan (Hyaluronate, Hyaluronic acid)	N-Acetyl-D-Glucosamine (N-Acetylglucosamine), D-Glucuronic acid (D-Glucuronate)	
Mucin (Mucus Glycoprotein)	D-Galactose, N-Acetyl-D-Glucosamine (N-Acetylglucosamine), D-Glucuronic acid (D-Glucuronate), N-Acetylneuraminic acid (N-acetylneuraminate, Neu5Ac, Sialic acid), D-Galacturonate, L-Fucose, N-Acetylgalactosamine (N-Acetyl-D-galactosamine), L-Serine (Serine, D-Serine), L-Cysteine (Cysteine, D-Cysteine), L-Proline (Proline, D-Proline), L-Threonine (Threonine), Sulfate (Sulfuric acid, Sulfite)	
Pectin	D-Galacturonate	
Starch (Amylopectin, Amylose, 1,4-alpha-D-Glucan, Pullulan, Resistant starch, Glycogen)	D-Glucose (Glucose), Maltose, Dextrin (Maltotriose, Maltodextrin, Maltohexaose, Maltotetraose, Maltooligosaccharide)	
Triglyceride	1,2-diacylglycerol (1-acylglycerol, Monoacylglycerol, Monoglyceride, Monoacylglycerol, Diacylglycerol), Glycerol	
Xylan	D-Xylose (Xylose), XOS (Xylooligosaccharide)	
Polypeptide	L-Alanine (D-Alanine, Alanine), L-Arginine (Arginine), L-Asparagine (Asparagine), L-Aspartate (Aspartate, D-Aspartate), L-Cysteine (Cysteine, D-Cysteine), L-Glutamine (D-Glutamine, Glutamine), L-Leucine (Leucine), L-Histidine (Histidine), L-Isoleucine (Isoleucine), L-Threonine (Threonine), L-Lysine (Lysine, D-Lysine), L-Methionine (D-Methionine), L-Phenylalanine (Phenylalanine, D-Phenylalanine), L-Proline (Proline, D-Proline), L-Serine (Serine, D-Serine), L-Tryptophan (Tryptophan), L-Tyrosine (Tyrosine), L-Valine (Valine, D-Valine), L-Glycine (Glycine), L-Glutamate (L-Glutamic acid, Glutamate, D-Glutamate), Prolylglycine, Glycyl-Phenylalanine, Leucyl-leucine, Alanylalanine, Glycylproline, Glycyl-leucine, Leucylglycine, Glycylglycine	Polypeptides can be broken down into amino acids and small peptides. Among them, only the compounds which have associations with the microbial species or the host cells in NJC19 are listed as the degradation products in Column B.
Arabinan	D-Arabinose (L-Arabinose, Arabinose, L-Arabinopyranose, L-Arabinofuranose)	
Mannan	D-Mannose (Mannose)	
DNA RNA Polynucleotide	Adenine, Cytosine, Guanine, Thymine, Uracil	
