# Publisher Correction: Large-scale metabolic interaction network of the mouse and human gut microbiota

**DOI:** 10.1038/s41597-020-00615-x

**Published:** 2020-08-12

**Authors:** Roktaek Lim, Josephine Jill T. Cabatbat, Thomas L. P. Martin, Haneul Kim, Seunghyeon Kim, Jaeyun Sung, Cheol-Min Ghim, Pan-Jun Kim

**Affiliations:** 1 Department of Biology, Hong Kong Baptist University, Kowloon, Hong Kong; 2 School of Life Sciences, Ulsan National Institute of Science and Technology, Ulsan, 44919 Republic of Korea; 3 Analytics, computing, and complex Systems Laboratory, Asian Institute of Management, Makati, 1229 Metro Manila Philippines; 4 Department of Physics, Ulsan National Institute of Science and Technology, Ulsan, 44919 Republic of Korea; 5 Department of Physics, Pohang University of Science and Technology, Pohang, Gyeongbuk 37673 Republic of Korea; 6 Samsung SDS, Seoul, 05510 Republic of Korea; 7 Microbiome Program, Center for Individualized Medicine, Mayo Clinic, Rochester, MN 55905 USA; 8 Division of Surgical Research, Department of Surgery, Mayo Clinic, Rochester, MN 55905 USA; 9 Division of Rheumatology, Department of Internal Medicine, Mayo Clinic, Rochester, MN 55905 USA; 10 Department of Molecular Pharmacology and Experimental Therapeutics, Mayo Clinic, Rochester, MN 55905 USA; 11 Center for Quantitative Systems Biology, Hong Kong Baptist University, Kowloon, Hong Kong; 12 Institute of computational and Theoretical Studies, Hong Kong Baptist University, Kowloon, Hong Kong; 13 Abdus Salam International Centre for Theoretical Physics, 34151 Trieste, Italy

Correction to: *Scientific Data* 10.1038/s41597-020-0516-5, published online 26 June 2020

Following publication of this Data Descriptor, a number of errors were noted in the manuscript text, Table 1, Online-only Table 5 and Supplementary Table 1 in relation to a formatting change made to Supplementary Table 1.

Erroneous linkage to Figures 2 and 3 in the Figure 3 legend have been removed.

In addition, Figure 1 has been replaced to include an additional motif that had been excluded at publication.

These errors have been corrected in both the HTML and PDF versions of this Data Descriptor.

